# Discovery of NKCC1 as a potential therapeutic target to inhibit hepatocellular carcinoma cell growth and metastasis

**DOI:** 10.18632/oncotarget.20240

**Published:** 2017-08-12

**Authors:** Yaya Zhou, Wei Sun, Ning Chen, Chen Xu, Xinxin Wang, Kun Dong, Binxue Zhang, Jian Zhang, Ning Hao, Aihua Sun, Handong Wei, Fuchu He, Ying Jiang

**Affiliations:** ^1^ State Key Laboratory of Proteomics, National Center for Protein Sciences Beijing, Beijing Proteome Research Center, Beijing Institute of Radiation Medicine, Beijing 102206, P. R. China; ^2^ Department of Pathology, Beijing Youan Hospital of Capital Medical University, Beijing 100069, P. R. China; ^3^ Institutes of Biomedical Sciences, Fudan University, Shanghai 200032, P. R. China

**Keywords:** hepatocellular carcinoma, metastasis, plasma membrane protein, NKCC1, therapeutic target

## Abstract

Metastasis is the essential cause for the high mortality of hepatocellular carcinoma (HCC). In order to investigate the mechanism of metastasis, and to discover therapeutic targets for HCC, the quantitative proteomic technique was applied to characterize the plasma membrane proteins of two HCC cell lines with low (MHCC97L) or high (MHCC97H) metastatic potentials. One of the plasma membrane proteins, sodium-potassium-chloride cotransporter 1 (NKCC1), was upregulated in MHCC97H cell line. Immunohistochemistry result in HCC patients showed that NKCC1 expression was associated with poor differentiation and microvascular invasion. Knockdown of NKCC1 via RNA interference reduced HCC cell proliferation and invasion abilities *in vitro* and *in vivo*, whereas over-expression of NKCC1 significantly increased HCC cell proliferation and invasion abilities *in vitro* and *in vivo*. Additionally, blocking NKCC1 activity with bumetanide attenuated the proliferation and invasion abilities of HCC cells *in vitro* and limited the HCC growth *in vivo*. Further results suggested that NKCC1 promotes the invasion ability via MMP-2 activity, and that the WNK1/OSR1/NKCC1 signal pathway might play roles in HCC metastasis. For the first time, our study demonstrated that NKCC1 plays a role in HCC metastasis, and could be served as a potential target to inhibit HCC cell growth and metastasis.

## INTRODUCTION

Hepatocellular carcinoma (HCC) is one of the most common cancers worldwide [1]. Metastasis is the main cause for poor prognosis and high fatality rates in HCC [2]. Because of a high incidence of recurrence and metastasis, the overall survival of patients with HCC after surgery remains unsatisfactory [3]. For the last decade, although many molecules associated with HCC metastasis have been identified, such as AFP, CK19, VEGF, TGF-β1 and HSP70 [4], the mechanism of HCC metastasis is still not well known. Therefore, a detailed investigation of the mechanism of metastasis and the discovery of reliable therapeutic targets are urgently needed.

Plasma membrane proteins are associated with multiple steps in the process of metastasis, such as breaking away from the primary site [5], adhesion to the extracellular matrix [6], infiltration into blood and lymphatic vessels [7], cell migration [8], and lodging in target organs. Furthermore, plasma membrane proteins are major targets for protein-based drug discovery [9–11]. Multiple functional receptors and ion channels have been approved as valid drug targets for cancer therapy [12–14], such as growth factor receptors [12, 15], cytokine receptors [16], potassium channels [14, 17] and so on. Despite their importance in cancer research, the analysis of plasma membrane proteins remains challenging due to difficulties in their isolation or enrichment. In this study, we used the colloidal silica pellicle technique [18], which is based on the ionic interaction of a positively-charged solid support with the cell surface, to separate the plasma membrane from intact HCC cells. Purity evaluation of the plasma membrane by transmission electron microscopy and Western blotting demonstrated significant enrichment of plasma membrane proteins with little contamination from other cellular organelles.

**Figure 1:**
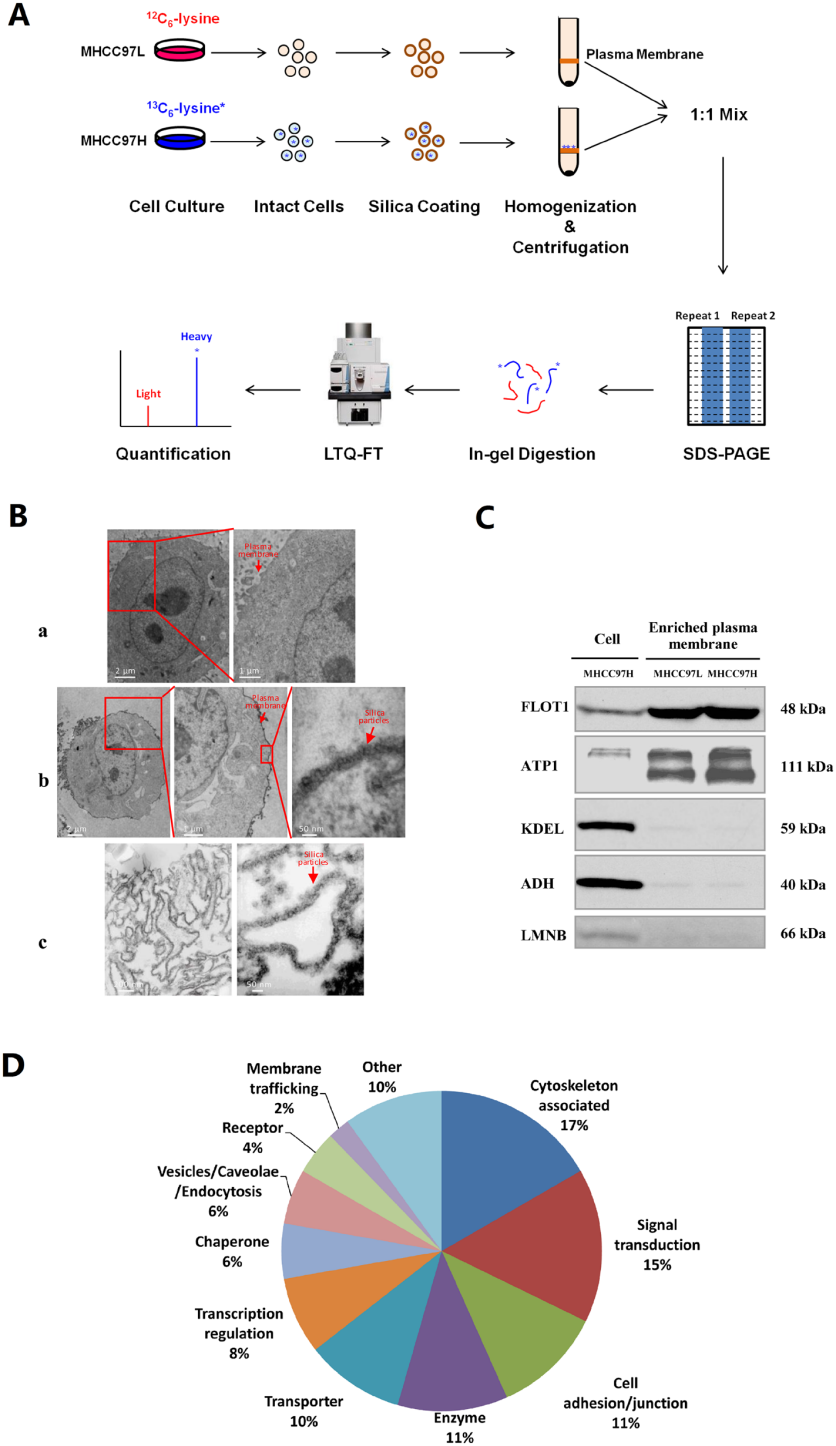
Proteome analysis of plasma membrane proteins from MHCC97L and MHCC97H cells **(A)** Schematic showing the process of plasma membrane separation and SILAC quantitative analysis in the MHCC97L and MHCC97H cell lines. **(B)** Electron micrographs of an uncoated cell (B-a), a coated cell (B-b), and isolated plasma membrane sheets (B-c). **(C)** Purity analysis of plasma membrane proteins by Western blotting in enriched plasma membrane and whole cell lysates, using the plasma membrane marker flotillin-1 (FLOT1), the endoplasmic reticulum marker KDEL, the cytosolic marker alcohol dehydrogenase (ADH) and the nuclear marker lamin B (LMNB). **(D)** Functional distribution of differentially expressed proteins localized in the plasma membrane.

Stable-isotope labeling by amino acids in cell culture (SILAC) is a metabolic labeling strategy that is compatible with the quantitative analysis of hydrophobic proteins and alkaline proteins, including membrane protein identification and quantification [19]. We used SILAC to analyze plasma membrane proteins isolated from the MHCC97L and MHCC97H cell lines (Figure 1A). These two cell lines originate from the same cell line MHCC97 and present low (MHCC97L) or high (MHCC97H) metastatic abilities [20], which are good models for HCC metastasis study. The whole cell proteomes of these two cells have been analyzed in previous studies [21, 22]. However, we’d like to focus on the plasma membrane in this study. Bumetanide-sensitive sodium-potassium-chloride cotransporter 1 (NKCC1), which was found upregulated in MHCC97H whole cell lysate in our previous study, was found upregulated again in the plasma membrane. NKCC1 localizes in the plasma membrane, and participates in the reabsorption of Na^+^, K^+^ and Cl^-^, regulating the volume of the liver cell [23]. The dysregulation of NKCC1 was shown to be associated with meningioma [24] and glioma [25, 26]. In addition to the *in vitro* experiments on HCC cell lines, we further confirmed the upregulation of NKCC1 in human HCC tissues with poor differentiation and microvascular invasion. However, the mechanism of NKCC1 mediated HCC metastasis was not illustrated before. Considering the potential important role NKCC1 plays in HCC metastasis, functional validation of NKCC1 was performed by overexpression, RNA interference (RNAi) and activity blocking with the inhibitor *in vitro* and *in vivo*, demonstrating the positive association of NKCC1 with the proliferation and invasion abilities of HCC cells. Further results on the downstream regulation of the activity of matrix metalloproteinase 2 (MMP-2) and the phospho-activation of NKCC1 mediated by the upstream kinases with-no-K (Lysine) kinase 1 (WNK1) and oxidative stress-responsive kinase 1 (OSR1) shed light on the molecular mechanism involving NKCC1 in HCC metastasis. Our study suggested that NKCC1 might be a potential therapeutic target to inhibit HCC growth and metastasis.

## RESULTS

### Enrichment and purity evaluation of plasma membrane proteins

The colloidal silica pellicle technique is based upon the ionic interaction of the cell surface with a positively-charged solid support. After homogenization, the coated plasma membrane can be enriched by density gradient centrifugation due to its high density. We evaluated the purity and the enrichment efficiency of plasma membrane by transmission electron microscopy and Western blotting. The electron micrographs showed that the cell and the subsequently isolated plasma membrane were successfully coated by the silica pellicle, whereas the control cells remained uncoated (Figure 1B). The silica pellicle uniformly adhered to the outer surface of the cell, without coating the organelles inside the cell.

A number of subcellular organelle marker protein antibodies, including flotillin-1 (plasma membrane), KDEL (endoplasmic reticulum), alcohol dehydrogenase (cytosol), and lamin B (nucleus) were also used for Western blotting to evaluate the enrichment of plasma membrane and the contamination from other subcellular organelles in the extracts (Figure 1C). It was found that the plasma membrane was highly enriched in the isolated fractions, with little contamination from other organelles. Since the levels of enrichment and contamination were similar in MHCC97L and MHCC97H, the purified plasma membranes from these two cell lines could be mixed to characterize differentially expressed proteins.

### Protein identification and quantification

SILAC analysis of plasma membrane proteins from HCC cell lines with low (MHCC97L, labeled with ^12^C_6_ lysine) and high (MHCC97H, labeled with^13^C_6_ lysine) metastatic potentials identified 2070 proteins in total, among which quantification information was available for 1181 proteins. Supplementary Figure 1 shows the SILAC ratio distribution for all the proteins quantified. The log_2_-ratios of most proteins were distributed around 0, indicating that the proteins of MHCC97L and MHCC97H were mixed equally and that MHCC97H was efficiently labeled. A cutoff ratio of 1.68 was calculated from 1 + (2.5 × median absolute deviation) [27]. There were 114 upregulated proteins and 76 downregulated proteins in MHCC97H compared with MHCC97L (Supplementary Table 1).

### Analysis of differentially expressed proteins

**Figure 2:**
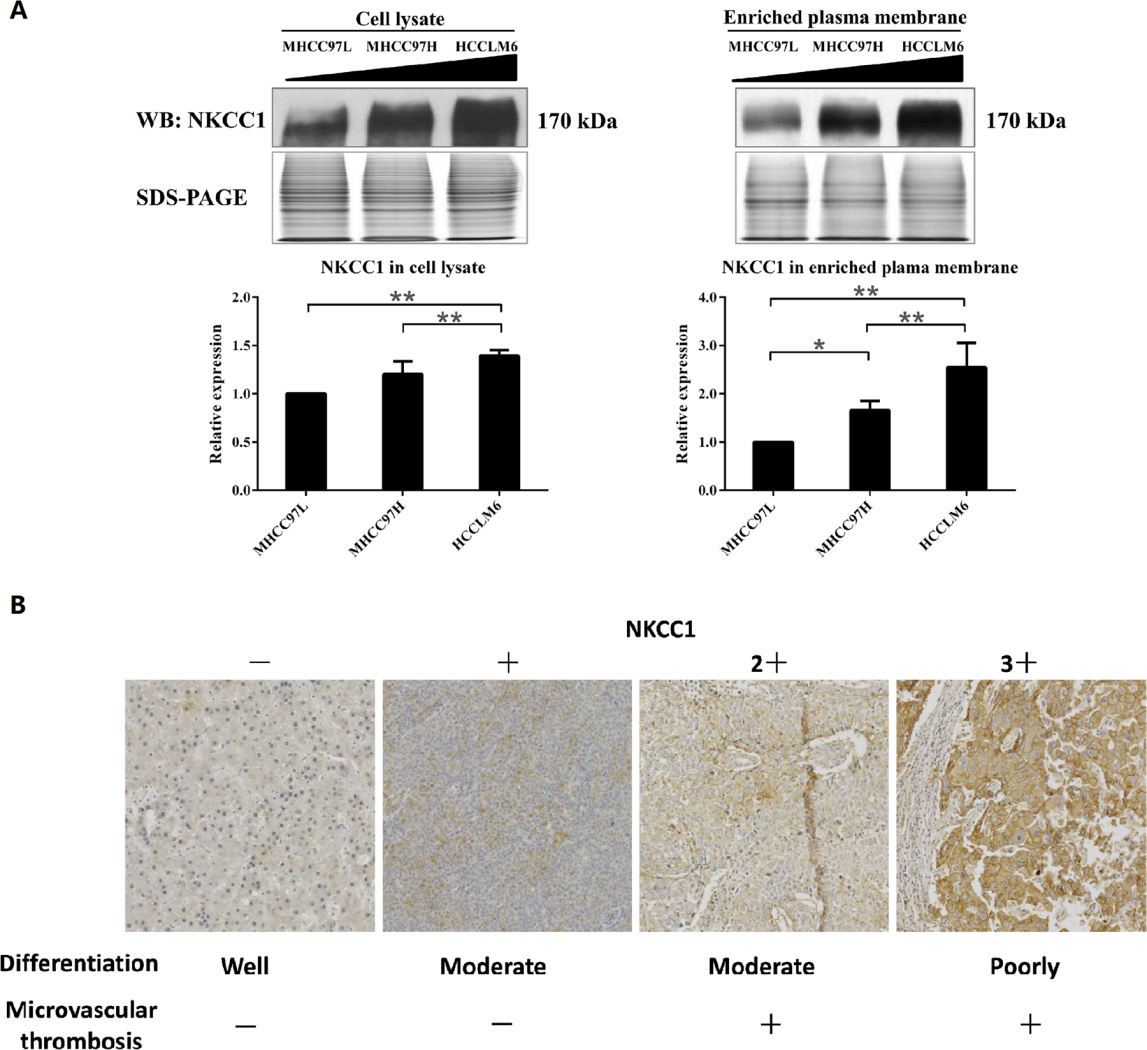
Detection of NKCC1 in MHCC97 cell lines and HCC tissues **(A)** Western blotting results validated the upregulation of NKCC1 in highly metastatic HCC cell lines. The triangle indicates the sequentially increased metastatic abilities of the MHCC97L, MHCC97H and HCCLM6 cell lines. * *p*<0.05 or ** *p*<0.01 indicates a significant difference of NKCC1 expression level between HCC cell lines (one-way ANOVA). **(B)** The representative immunohistochemistry staining results of NKCC1 in HCC tumor tissue. (F-a) Result of sample W2 as the representative staining of (-). Weak and partial plasma membrane staining in ≤10% cancer cells. (F-b) Result of sample C7 as the representative staining of (1+). Weak and partial plasma membrane staining in >10% cancer cells. (F-c) Result of sample C1 as the representative staining of (2+). Moderate and partial plasma membrane staining in >10% cancer cells, or strong and complete plasma membrane staining in ≤10% cancer cells. (F-d) Result of sample C9 as the representative staining of (3+). Strong and complete plasma membrane staining in >10% cancer cells.

Of the 190 differentially expressed proteins, 90 proteins (47%) were localized to the plasma membrane, according to Gene Ontology (GO, http://geneontology.org/), Human Protein Atlas (HPA, http://www.proteinatlas.org/), Uniprot (http://www.uniprot.org/) and literature annotation (Supplementary Table 1). These 90 differentially expressed plasma membrane proteins included cytoskeleton-associated proteins, signal transduction proteins, cell adhesion/junction molecules, enzymes, transporters, and others (Figure 1D). In an earlier study by our group, NKCC1 was detected to be upregulated in the whole cell lysate of highly metastatic HCC cell lines and the sera of metastatic HCC patients [22], but the mechanism of NKCC1 mediated metastasis was not intensively illustrated. The upregulation of NKCC1 in MHCC97H plasma membrane indicated again that this protein may promote HCC metastasis. Therefore, we decided to focused on the functional investigation of NKCC1 in HCC metastasis. The upregulation of NKCC1 in whole cell lysates and enriched plasma membranes were further validated by Western blotting from three cell lines with progressively increased metastatic potentials (MHCC97L<MHCC97H<HCCLM6) (Figure 2A). The SILAC quantification results of NKCC1 are shown in Supplementary Figure 2.

### Correlation between NKCC1 expression level and clinical features in HCC

NKCC1 expression levels in HCC tumor tissues were detected in 67 cases by immunohistochemistry staining (Figure 2B). Using Chi-Square test (with SPSS version 16.0 software), tumor differentiation and microvascular invasion were shown to have significant associations with NKCC1 expression (*p*<0.05, Table 1), while no significant association between NKCC1 expression and gender, age, glypican-3, keratin 19 or Ki-67.

**Table 1:** Correlation between NKCC1 expression and clinical features in HCC

Clinical features		Cases	NKCC1		Contingency coefficient	p value
			Negative	Positive		
Gender	Male	51	16	35	0.163	0.175
	Female	16	8	8		
Age	<60	51	19	32	0.053	0.662
	≥60	16	5	11		
Tumor Differentiation	Poorly	26	5	21	0.266	0.024^*^
	Well, moderate	41	19	22		
Microvascular tumor thrombus	Negative	42	19	23	0.247	0.037^*^
	Positive	25	5	20		
Glypican-3	Negative	8	3	5	0.013	0.916
	Positive	59	21	38		
Keratin 19	Negative	45	18	27	0.124	0.308
	Positive	22	6	16		
Ki-67	Low (<20%)	48	19	29	0.092	0.455
	High (≥20%)	17	5	12		

*p < 0.05 indicates a significant difference between NKCC1 expression and the clinical features, according to Chi-Square test.

### Upregulation of NKCC1 promotes cell proliferation and invasion *in vitro*

**Figure 3:**
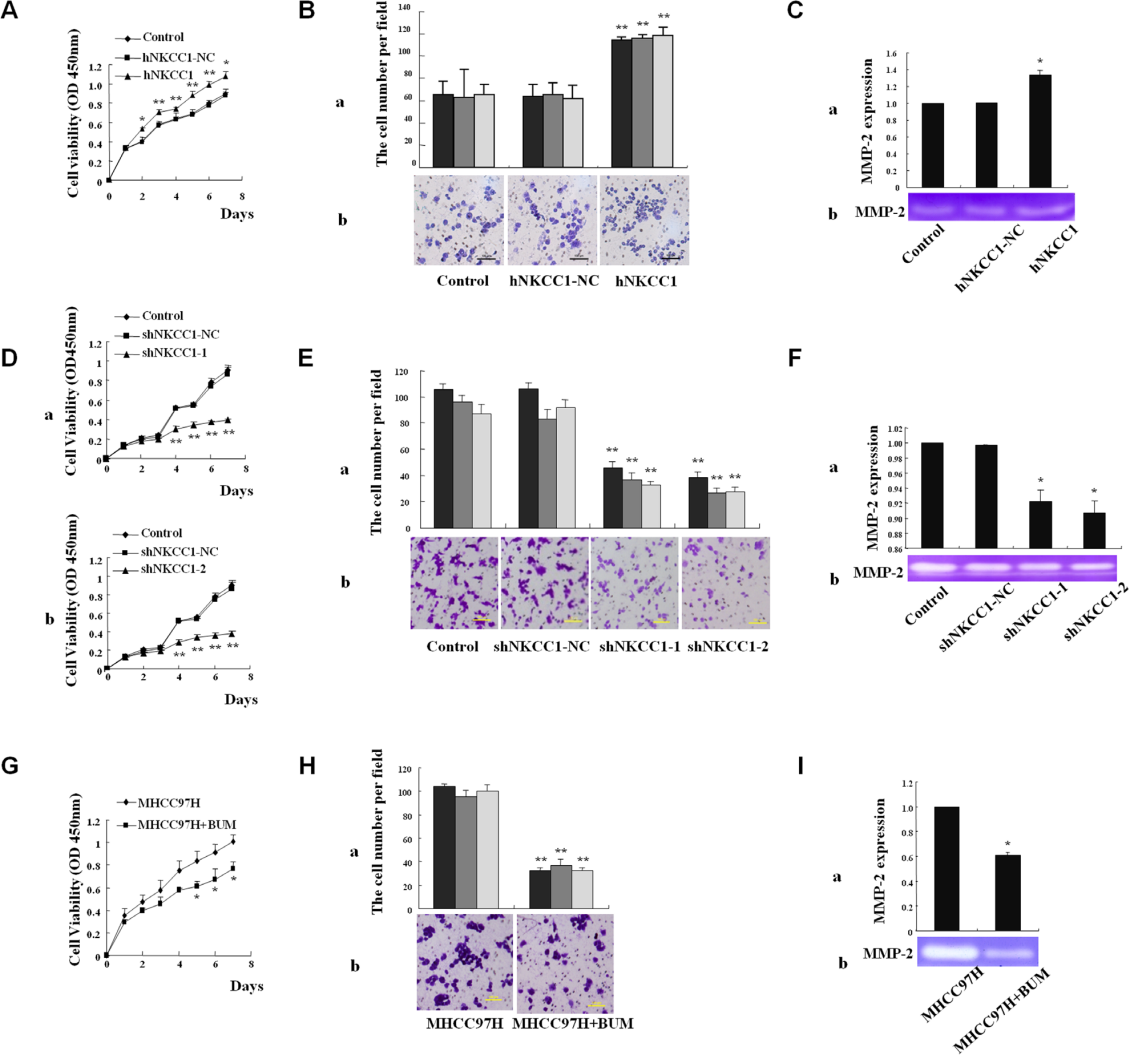
Effects of NKCC1 overexpression/knockdown and inhibitor treatment on HCC cell growth, invasion, and MMP-2 activity *in vitro* **(A)** CCK-8 kit analysis of cell viability shows that MHCC97L cell proliferation was significantly promoted upon NKCC1 overexpression. **(B)** The Matrigel assay shows that the invasion of MHCC97L cells was significantly promoted upon NKCC1 overexpression. (B-a) Counts of trespassed cells per field (from at least five fields) from three experiments (mean±SD). (B-b) Representative photographs after 24-h incubation. **(C)** The activity of MMP-2 in MHCC97L cells was significantly increased after NKCC1 overexpression. (C-a) Statistical results of triplicate experiments. (C-b) Representative photographs. **(D)** CCK-8 kit analysis shows that MHCC97H cell proliferation was significantly inhibited upon NKCC1 knockdown. (D-a) Knockdown with shNKCC1-1. (D-b) Knockdown with shNKCC1-2. **(E)** The Matrigel assay shows that the invasion of MHCC97H cells was significantly inhibited upon NKCC1 knockdown. (E-a) Counts of trespassed cells per field (from at least five fields) from three experiments (mean±SD). (E-b) Representative photographs after 24-h incubation. **(F)** The activity of MMP-2 in MHCC97H cells was significantly inhibited upon NKCC1 knockdown. (F-a) Statistical results of triplicate experiments. (F-b) Representative photographs. **(G)** CCK-8 kit analysis shows that MHCC97H cell proliferation was significantly inhibited following bumetanide treatment. **(H)** The matrigel assay shows that the invasion of MHCC97H cells was significantly inhibited following bumetanide treatment. (H-a) Counts of trespassed cells per field (from at least five fields) from three experiments (mean±SD). (H-b) Representative photographs after 24-h incubation. **(I)** The activity of MMP-2 in MHCC97H cells was significantly inhibited following bumetanide treatment. (I-a) Statistical results of triplicate experiments. (I-b) Representative photographs. Statistical significance was determined with one-way ANOVA (A, B, C, D, E and F) or two-tailed Student's *t*-test (G, H and I). * *p*<0.05 or ** *p*<0.01 indicates a significant difference between NKCC1 overexpression/knockdown or inhibitor treatment with the normal control group. hNKCC1, overexpression of NKCC1; NC, negative control; shNKCC1, knockdown of NKCC1 with shRNA; BUM, bumetanide treatment.

Mammalian expression vectors containing NKCC1 were stably transfected into the MHCC97L cell line, which has low endogenous expression of NKCC1 (Supplementary Figure 3). NKCC1 overexpression promoted cell proliferation (analyzed using the CCK-8 kit, Figure 3A) and invasion (analyzed using the Transwell assay, Figure 3B), compared to control cells transfected with empty pcDNA3.1 vector. The activity of matrix metalloproteinase 2 (MMP-2) was detected by a gelatin-based zymography assay. NKCC1 overexpression significantly promoted the activity of MMP-2 in MHCC97L cells (Figure 3C). The experiments were repeated three times, and the average values of triplicate tests with standard deviations are represented.

### Downregulation of NKCC1 inhibits cell proliferation and invasion *in vitro*

Two short hairpin RNAs (shRNAs) that target NKCC1 (shRNA-1 and shRNA-2) efficiently knocked down endogenous NKCC1 expression in MHCC97H cells, as shown in Supplementary Figure 4. Stable knockdown of NKCC1 inhibited the proliferation of MHCC97H cells (Figure 3D), whereas cell apoptosis under conditions of serum starvation was not obviously influenced compared to control cells transfected with shRNA-NC (data not shown). Further results showed that the silencing of NKCC1 significantly inhibited the invasion (Figure 3E) of MHCC97H cells *in vitro* compared to control cells. These results suggest that NKCC1 contributes to metastasis with a significant effect on the proliferation and invasion of MHCC97H cells. We also found that downregulation of NKCC1 significantly inhibited the activity of MMP-2 in MHCC97H cells (Figure 3F).

### Blocking NKCC1 activity with bumetanide diminishes cell proliferation and invasion *in vitro*

Bumetanide, a NKCC1 activity inhibitor, was applied to analyze the role of NKCC1 in HCC metastasis. The enzyme activity of NKCC1 depends on phosphorylation. Therefore, we first demonstrated that treatment with 50 μM of bumetanide could inhibit NKCC1 activity in HCC cell lines MHCC97H and Huh7, using fluorescence intensity measurement of enzyme activity (Supplementary Figure 5A, Supplementary Figure 5C) and detection of phosphorylated NKCC1 (Supplementary Figure 5B, Supplementary Figure 5D). Subsequently, we showed that blocking NKCC1 activity via bumetanide could diminish cell proliferation (Figure 3G, Supplementary Figure 6A) and invasion (Figure 3H, Supplementary Figure 6B), and inhibit the activity of MMP-2 (Figure 3I, Supplementary Figure 6C). These results indicate that NKCC1 activity is associated with the metastatic ability of HCC cells *in vitro*.

### NKCC1 enhances HCC growth *in vivo*

**Figure 4:**
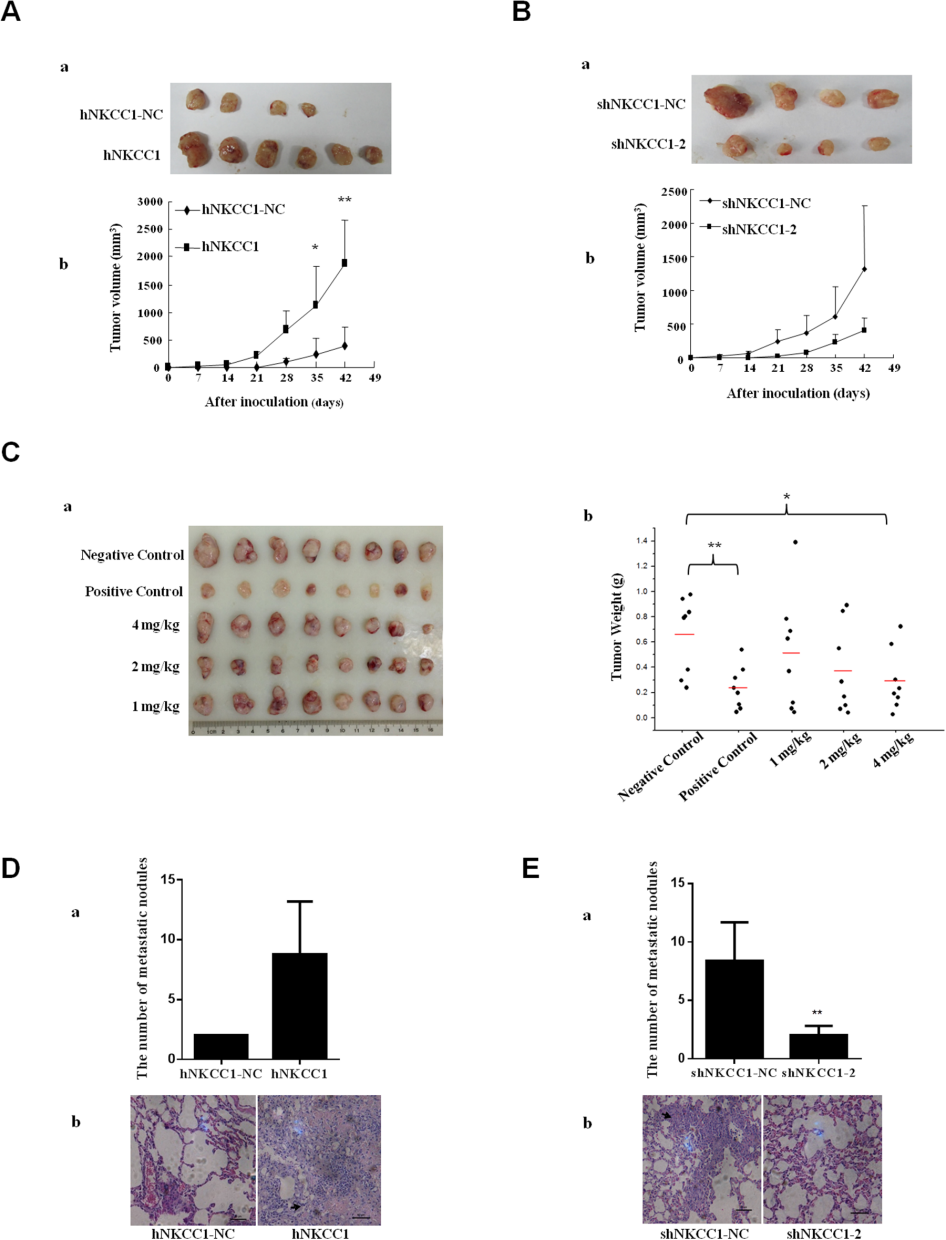
Effects of NKCC1 overexpression/knockdown and inhibitor treatment on the growth and extrahepatic metastasis of HCC cells *in vivo* **(A)** Sizes of tumors formed from subcutaneously injected NKCC1-overexpressing MHCC97L cells and control MHCC97L cells transfected with empty vector. **(B)** Sizes of tumors formed from subcutaneously injected NKCC1 knockdown MHCC97H cells and control MHCC97L cells transfected with shRNA-NC. Tumor growth was monitored every week by measuring the tumor diameter (mean±SD). (A-a) and (B-a) Photographs of all xenograft tumors in mice. (A-b) and (B-b) Statistical results of tumor volume. **(C)** The effect of bumetanide on MHCC97H cell xenotransplantation *in vivo*. Water (negative control), 350 mg/kg of sorafenib (positive control), or bumetanide (1 mg/kg, 2 mg/kg, or 4 mg/kg) were administered by oral gavage. Tumor size (C-a) and tumor weight (C-b) in MHCC97H subcutaneously inoculated nude mice are shown (bars indicate average value). **(D)** NKCC1-overexpressing MHCC97L and control cells or **(E)** NKCC1-knockdown MHCC97H and control cells were injected into the tail veins of nude mice. Lung metastasis was checked after 16 weeks. (D-a) The number of the lung metastatic nodules in NKCC1 overexpression group was compared with that in the control group. (D-b) Histological images of lung metastatic nodules formed from MHCC97L cells. (E-a) The number of lung metastatic nodules in the NKCC1 knockdown group was compared with that in the control group. (E-b) Histological images of lung metastatic nodules formed from MHCC97H cells. * *p*<0.05 or ** *p*<0.01 indicates a significant difference (two-tailed Student's *t*-test). hNKCC1, overexpression of NKCC1; NC, negative control; shNKCC1, knockdown of NKCC1 with shRNA.

To extend the above results to an *in vivo* context, we subcutaneously injected MHCC97L cells (2×10^6^) stably transfected with mammalian expression vectors containing NKCC1, or control cells transfected with empty vector, into six BALB/c nude mice. After six weeks, it was observed that the sizes of tumors formed from NKCC1-overexpressed cells were significantly increased compared to the tumor sizes from control cells (Figure 4A). These results suggest that upregulation of NKCC1 could promote HCC growth.

We also subcutaneously injected stable NKCC1-knockdown MHCC97L cells or control cells transfected with shRNA-NC (2×10^6^) into six BALB/c nude mice. The average size of tumors formed from NKCC1-knockdown cells was smaller compared to that of tumors from control cells after 6 weeks (Figure 4B).

### Bumetanide inhibits HCC growth *in vivo*

After 18 days of administration, the tumor weights in 4 mg/kg bumetanide treated mice were smaller (*p*<0.05) than those in the negative control group; however, the difference was not as significant as that of the sorafenib-treated positive control group (*p*<0.01, shown in Figure 4C). None of the mice died during the process, indicating the safety of drug administration. The major side effect due to the use of 4 mg/kg bumetanide was body weight loss, while no obvious body weight changes were detected in 1 mg/kg or 2 mg/kg bumetanide treated mice (Supplementary Table 2). This adverse effect might due to the loss of water and electrolytes after using too much bumetanide.

### NKCC1 contributes to tumor metastasis *in vivo*

To further explore the effect of NKCC1 on tumor metastasis *in vivo*, we injected NKCC1-overexpressed MHCC97L cells and control cells transfected with empty vector (2×10^6^ cells) into the tail veins of nude mice, and checked extrahepatic metastasis in the lungs after 16 weeks. On average, more metastases were found in the lungs of mice injected with NKCC1-overexpressed MHCC97L cells (Figure 4Da). This experiment was also performed with NKCC1-knockdown MHCC97H cells and control cells transfected with shRNA-NC. The number of metastatic tumors in the lungs significantly decreased in the knockdown group compared with control (Figure 4Ea). The presence of lung tumors was confirmed by histological analysis (Figure 4Db and Figure 4Eb). Tumors formed in the liver could also be found in these mice (Supplementary Figure 7). These data suggest that NKCC1 plays a crucial role in metastasis of HCC cells *in vivo*.

The effects of NKCC1 knockdown on intrahepatic metastasis *in vivo* were also evaluated. We injected stable NKCC1-knockdown MHCC97H cells, cells transfected with shRNA-NC, or control MHCC97H cells (2×10^6^ cells), into the spleens of BALB/c nude mice. After 8 weeks, obvious liver metastatic nodules could be seen in mice inoculated with MHCC97H cells or cells transfected with shRNA-NC (Supplementary Figure 8A). However, the total liver weight was significantly decreased in groups inoculated with NKCC1-knockdown MHCC97H cells than with shRNA-NC (Supplementary Figure 8B). This result suggests that NKCC1 knockdown inhibited the intrahepatic metastasis of HCC cells in nude mice. The presence of tumors in the liver was confirmed by histological analysis (Supplementary Figure 8C).

### Protein levels of WNK1/OSR1/NKCC1 in liver cells are positively associated with metastatic ability

**Figure 5:**
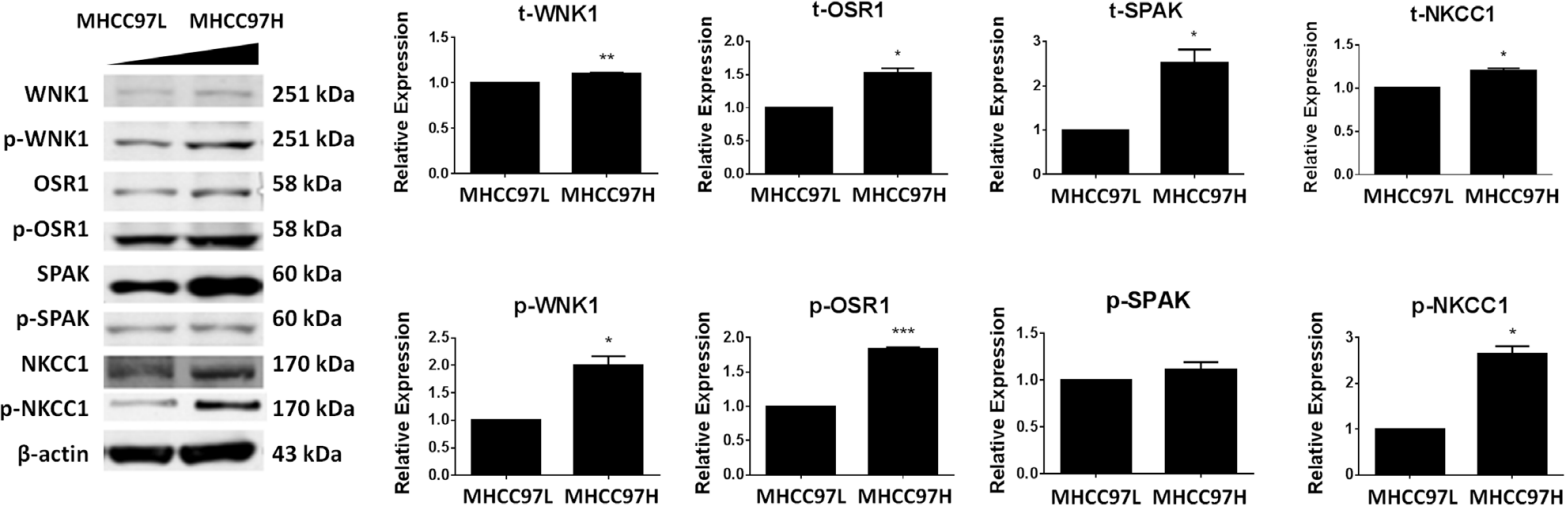
Expression levels of WNK1, OSR1, SPAK and NKCC1 in MHCC97L and MHCC97H cells The total and phosphorylated protein levels of WNK1, OSR1, SPAK, and NKCC1 were detected by Western blotting in HCC cell lines with sequentially increased metastatic abilities (MHCC97L<MHCC97H). Total protein levels (t-) and active phosphorylated protein levels (p-) of WNK1, OSR1, and NKCC1 were all significantly increased in MHCC97H. The total protein level of SPAK was significantly increased in MHCC97H, but the active phosphorylated protein level of SPAK remained unchanged. * *p*<0.05, ** *p*<0.01 or *** *p*<0.001 indicates a significant difference (two-tailed Student's *t*-test).

Total and phosphorylated protein levels of NKCC1 and three upstream kinases WNK1, OSR1, and SPS1-related proline/alanine-rich kinase (SPAK) were detected by Western blotting in HCC cell lines with different metastatic abilities (MHCC97H>MHCC97L). The result showed that the total expression levels of NKCC1, WNK1, OSR1, and SPAK were positively associated with metastatic ability. The same result was obtained for the active phosphorylated protein levels of the above proteins, with the exception of SPAK (Figure 5).

## DISCUSSION

HCC metastasis is closely related to multiple factors, such as cancer cell adhesion and motility, extracellular matrix degradation, body immunity, tumor angiogenesis, and other processes. Plasma membrane proteins are associated with multiple steps of such processes. A direct investigation of plasma membrane proteins, especially dynamically changed adhesion molecules, signal transduction pathway component proteins, transporters, and enzymes on the tumor cell surface, can help elucidate the mechanisms underlying tumor metastasis and discover potential biomarkers or therapeutic targets.

In this study, plasma membranes from HCC cell lines were highly enriched by colloidal silica pellicle technique (Figure 1C). After SILAC, which facilitated the quantitative proteome analysis of plasma membrane proteins from HCC cell lines with low or high metastatic potential (MHCC97L and MHCC97H), 2070 reliable proteins were identified in total. According to the GO, HPA, Uniprot, and literature annotations, 90 (47%) of these 190 dysregulated proteins (Supplementary Table 1) showed plasma membrane localization, and mainly involved in cytoskeleton association, signal transduction, cell adhesion/junction, catelyzation, transportation and so on (Figure 1D). Some of these proteins are well known to be involved in metastasis, such as carcinoembryonic antigen-related cell adhesion molecule 6 (CEACAM6) [30], platelet endothelial cell adhesion molecule 1 (PECAM-1) [31], heat shock proteins HSP 90-alpha [32] and HSP 90-beta [33]. Proteins reported to be involved in HCC metastasis associated were also identified. For example, tetraspanin-8 was found to be overexpressed in intrahepatic spreading HCC [34] and might be involved in hematogenous intrahepatic metastasis of liver cancer cells [35]. High mobility group protein B1 (HMGB1) could promote HCC cell line metastasis by activating RAGE signaling pathways [36] and inducing caspase-1 activation [37]. Hepatoma-derived growth factor (HDGF) was associated with apoptosis in metastatic HCC cells [38]. Integrin alpha-6 could mediate the metastasis of HCC cells through the phosphoinositide 3 kinase (PI3K)/adrenergic receptor kinase (ARK) and mitogen-activated protein kinase (MAPK)/extracellular signal-related kinase (ERK) pathways [39].

In addition to the proteins listed above, a series of important molecules that have not previously been linked to HCC metastasis were found to be dysregulated in this study. For example, CD109 (a negative modulating factor of transforming growth factor β1 [TGF-β1] signaling [40, 41]), sodium bicarbonate cotransporter 3 (which participate in the reabsorption of Na^+^), myristoylated alanine-rich C-kinase substrate (which binds to actin), and syntaxin 4 (which mediates matrix metalloproteinase secretion via promoting vesicle docking to the plasma membrane [42, 43]), were upregulated in the highly metastatic HCC cell line. On the other hand, peroxiredoxin 6, which may play a role in protection against oxidative injury, and voltage-dependent anion-selective channel protein 1, which is involved in cell volume regulation and apoptosis [44], were downregulated.

Solute carrier family 12 member 2, also known as bumetanide-sensitive sodium-potassium-chloride cotransporter 1 (NKCC1), was found to be upregulated in MHCC97H and the sera of metastatic HCC patients in an earlier study by our group [22]. The upregulation of NKCC1 in MHCC97H plasma membrane indicated again that this protein may play important role in HCC metastasis, besides the reported association with invasion in meningioma [26] and glioma [25]. However, the role of NKCC1 in HCC metastasis has not been functionally verified and elaborated.

So far, two distinct Na-K-Cl cotransporter isoforms have been identified, named NKCC1 and NKCC2. As shown in HPA, the NKCC1 isoform is expressed in a wide variety of tissues, whereas NKCC2 is found only in the kidney. Overexpression of NKCC1 promotes proliferation in normal fibroblast cells [45]. Inhibition of NKCC1 activity in airway smooth muscle cells facilitated cell apoptosis and decreased cell proliferation [46]. High expression and activity of NKCC1 was found in poorly differentiated gastric adenocarcinoma cells [47], esophageal squamous cell carcinoma [48], meningiomas [26], glioblastoma, and anaplastic astrocytoma tissues [49].

In our study, the upregulation of NKCC1 in the plasma membrane of a highly metastatic HCC cell line was further confirmed by Western blotting (Figure 2A). The NKCC1 expression levels in clinical samples were also detected by immunohistochemistry staining in tumor tissues from 67 HCC patients. Tumor differentiation and microvascular invasion were shown to have significant associations with the expression of NKCC1 (*p*<0.05, Table 1), keratin 19 and Ki-67 but not glypican-3 (Supplementary Table 3). The positive rate of NKCC1 in poorly differentiated HCC cases (21/26, 81%) was higher than that in well and moderate differentiated cases (23/42, 55%). The higher expression of NKCC1 in poorly differentiated tumor samples was also reported in other tumors such as sophageal squamous cell carcinoma [48], indicating that NKCC1 may be involved in tumor differentiation. In HCC patients with microvascular invasion, the positive rate of NKCC1 (20/25, 80%) was higher than that in patients without microvascular invasion (24/43, 56%). Existence of microvascular invasion is an indicator for higher metastasis rate. This result supported the positive relationship of NKCC1 with tumor metastasis. However, the expression of NKCC1 was not associated with gender, age, glypican-3, keratin 19 or Ki-67, indicating NKCC1 might be a potential independent prognosticator of HCC.

Based on the dysregulation of NKCC1 in metastatic HCC cell lines and its positive relationship with tumor metastasis in clinical HCC samples, overexpression, RNAi, and activity inhibition experiments were subsequently performed to investigate the relationship between NKCC1 expression and activity with the growth and metastatic ability of HCC cells and its potential value for clinical application.

We found that the overexpression of NKCC1 in MHCC97L cells with low metastatic ability significantly increased cell proliferation and invasion (Figure 3A and 3B), whereas RNAi knockdown of NKCC1 in MHCC97H cells with high metastatic ability significantly decreased cell proliferation and invasion (Figures 3D, and 3E). These results indicated that NKCC1 is associated with HCC growth and metastasis *in vitro*. Tumor xenograft assessed in subcutaneously inoculated nude mice showed that NKCC1 overexpression in MHCC97L cells significantly promoted tumor growth (Figure 4A), whereas NKCC1 knockdown in MHCC97H cells inhibited the growth of tumor on average (Figure 4B). These results indicate that NKCC1 is associated with HCC growth *in vivo*. Extrahepatic metastasis assessed in tail vein-injected nude mice demonstrated that NKCC1-overexpressing MHCC97L cells formed more metastatic tumor nodules in the lungs on average, whereas NKCC1-knockdown MHCC97H cells formed significantly fewer lung metastatic tumors, compared with control (Figure 4D and 4E). Intrahepatic metastasis assessed using spleen-inoculated nude mice suggested that NKCC1 knockdown significantly inhibited the formation of liver metastatic nodules in MHCC97H cells (Supplementary Figure 8). These results indicated that NKCC1 plays a crucial role in HCC extrahepatic and intrahepatic metastasis *in vivo*.

All the experiments above confirmed the positive association between NKCC1 expression and HCC growth and metastatic ability. Then we treated MHCC97H cells with the Food and Drug Administration (FDA)-approved NKCC1 inhibitor bumetanide. Bumetanide selectively binds to NKCCs and inhibits ion translocation of Na^+^, K^+^ and Cl^-^ [50, 51]. Considering that NKCC2 was only expressed in kidney and we only identified NKCC1 in MHCC cell lines, the inhibitor bumetanide should be selective for NKCC1 alone in MHCC cell lines in this study. As expected, blocking of NKCC1 activity with bumetanide significantly diminished cell proliferation and invasion *in vitro* (Figure 3G and 3H). *In vivo* experiments showed that 4 mg/kg bumetanide treatment for 18 days significantly inhibited the HCC growth (Figure 4C), although the inhibition effect was not as significant as that of sorafenib, a Food and Drug Administration (FDA)-approved anti-HCC drug used as the positive control. It has been proposed that ion channels and transporters could be promising targets for the treatment of cancer [52]. Our study demonstrates the therapeutic potential of the NKCC1 inhibitor bumetanide in HCC treatment.

After proving that the expression and activity of NKCC1 positively affected HCC growth and metastasis, we tried to investigate the mechanism of NKCC1 function in HCC metastasis. It was reported that NKCC1 modulated glioma cell migration through the regulation of focal adhesion dynamics and cell volume [49].

The WNK1/SPAK/OSR1 signaling pathway is a well-studied upstream regulatory component of NKCC1 [53], and it has been reported that WNK1 and OSR1 regulate the activation and phosphorylation of NKCC1 in human glioma cells [28, 29]. In this study, in HCC cell lines with different metastatic abilities, we detected the total and phosphorylated protein levels of NKCC1 and three upstream kinases, including WNK1 and its two substrates OSR and SPAK. We found that t-WNK1, t-OSR1, t-SPAK, t-NKCC1, p-WNK1, p-OSR1 and p-NKCC1 were positively associated with the metastatic ability in human HCC cells (Figure 5). The activation of p-WNK1, p-OSR1 and p-NKCC1 indicates that WNK1/OSR1/NKCC1 signaling pathway might play roles in HCC cell metastasis.

We also found that the activity of MMP-2 was significantly increased after NKCC1 overexpression (Figure 3C), or significantly reduced after NKCC1 knockdown (Figure 3F) or bumetanide treatment (Figure 3I, Supplementary Figure 6C), which explained another downstream mechanism of NKCC1 in HCC metastasis.

In conclusion, 190 differentially expressed proteins were identified after colloidal silica pellicle separation and SILAC quantitative analysis of plasma membrane proteins of HCC cell lines with low or high metastatic potentials. NKCC1 was further validated by Western blotting. *In vitro* and *in vivo* overexpression, RNAi, and activity inhibition experiments demonstrated that NKCC1 expression and activity positively affected the growth and metastatic ability of HCC cells, and underscored its potential value as HCC therapeutic target. Further mechanistic studies demonstrated that WNK1/OSR1-mediated phospho-activation of NKCC1 facilitates HCC metastasis. Furthermore, the activity of MMP-2 could also be regulated by NKCC1. Immunohistochemistry data in clinical samples indicated the correlation of NKCC1 expression with tumor differentiation and microvascular invasion. Our study is the first report on the role of NKCC1 in HCC growth and metastasis, and suggested its potential as a theraptic target of HCC.

### Experimental procedures

#### Cell culture

MHCC97L and MHCC97H cell lines were obtained from Liver Cancer Institute and Zhong Shan Hospital of Fudan University. They were cultured in L-lysine depleted high glucose DMEM (Haicheng Tiandi Science, Beijing, China) supplemented with 10% dialyzed fetal bovine serum (FBS) (PAA Laboratories, Linz, Austria), and either 0.1 mg/mL ^12^C_6_- (light labeled, MHCC97L) or ^13^C_6_- (heavy labeled, MHCC97H) L-lysine (98% purity; Cambridge Isotope Laboratories, Andover, MA, USA) at 37°C. After five rounds of cell doubling time, ^13^C_6_-L-lysine was efficiently incorporated into the cell proteome.

### Plasma membrane protein isolation by colloidal silica coating in living cells

About 8×10^7^ cells for each cell line were suspended in 15 mL of MES buffered saline (MBS, 20 mM MES, 0.15 M NaCl, pH 5), and then dispersed slowly into 150 mL of silica solution (2% silica, 20 mM MES, 0.15 M NaCl, pH 5). After mixing at 4°C for 10 min, the suspension was centrifuged at 300 g for 5 min and washed twice with MBS to remove the extra silica. The pellet was resuspended in 15 mL of MBS (pH 6), and then dispersed slowly into 150 mL of polyacrylic acid solution (0.1% polyacrylic acid, 20 mM MES, 0.15 M NaCl, pH 6). After mixing at 4°C for 10 min, the suspension was centrifuged at 300g for 5 min and washed twice with MBS. Then the coated cells were resuspended in 5 mL of homogenization buffer (10 mM HEPES, 250 mM sucrose, pH7.4, protease inhibitor Cocktail) and homogenized by Dounce homogenizer with B stick for 200 times. The lysates were centrifuged at 900 g for 10 min, then the pellet was resuspended in 2 mL of homogenization buffer and layered on 1.37 g/mL Nycodenz. The tubes were topped off with homogenization buffer. The silica coated plasma membrane sheets were pelleted at 28,000 g in an SW41Ti rotor for 60 min. The plasma membrane pellets on the interface were pooled and washed sequentially with 1 M KCl, 0.1 M Na_2_CO_3_ and PBS (pH 7.4), three times in each solution.

Then the plasma membrane proteins were dissolved in lysis buffer (4% SDS, 50 mM Tris-Cl, 100 mM DTT and protease inhibitor cocktail) followed by sonication and 95°C treatment for 5 min. After removal of the unsolvable particulate materials by centrifugation, protein concentrations were measured by RC DC protein assay and confirmed by SDS-PAGE. The purity and contamination were evaluated by transmission electron microscopy and Western blotting of organelle markers.

### SILAC quantification of plasma membrane proteins from MHCC97L and MHCC97H cell lines

Equal amounts (30 μg each) of plasma membrane proteins isolated by colloidal silica coating method from MHCC97L (^12^C_6_-lysine) and MHCC97H (^13^C_6_-lysine) cell lines were mixed and separated by a 12% SDS-PAGE gel. After Coomassie brilliant blue staining, each gel lane was cut into 46 bands for in-gel microwave-assisted trypsin digestion [54].

Peptides from each of the gel bands were analyzed by LTQ-FT mass spectrometer (Thermo Electron, San Jose, CA, USA) equipped with nanospray source and Agilent 1100 high-performance liquid chromatrography system (Santa Clara, CA, USA). The LTQ-FT was operated in the data-dependent mode. A full-scan MS experiment (m/z range from 400 to 2000) was acquired, followed by MS/MS on the top ten ions detected in the full-MS scan.

The raw data were analyzed with the searching algorithm implemented in the Thermo Proteome Discover (version 1.3), against a combined forward and reversed database of Human UniProt protein database (release date 20140319, 88703 entries) through local MASCOT search engine (version 2.3). Peptide abundances were calculated based on the areas of the monoisotopic peaks. Protein ratios were the average ratios of all quantified peptides. Two batches of cell line samples were used for SILAC experiment. In order to reduce random errors, proteins with ratio>1.68 (the average ratio of two repeat experiments) were considered as differentially expressed proteins, using a cutoff of 2.5 median absolute deviation [27]. The diagram of plasma membrane separation and SILAC quantitative analysis of MHCC97L and MHCC97H cell lines was shown in Figure 1A.

### Western blotting

Whole cell lysates and enriched membrane proteins of MHCC97L, MHCC97H and HCCLM6 cell lines were used for further confirmation by Western blotting. For phosphorylated protein detection, the cells were dissolved in lysis buffer containing 4% SDS, 5 mM NaF, 1 mM NaVO_3_, 2% DTT, 10 mM NaPPi, 120 mM Tris-Cl pH 6.8, and protease inhibitor Cocktail. Samples were loaded with an equal amount of protein per well, separated by SDS-PAGE and transferred to nitrocellulose membranes (Amersham Biosciences, UK). After blocking with 5% non-fat milk and 0.05% Tween-20 in PBS (PBST) for 1 h at room temperature, membrane was incubated with the antibody overnight at 4°C, followed by the horseradish peroxidase-conjugated secondary antibody (Zhongshanjinqiao Biotech Company, Beijing, China, 1:10000 dilution) for 1h at room temperature. Protein signals were detected by ECL kit (Pierce). Antibodies used for Western Blotting were shown in Supplementary Table 4.

### Immunohistochemistry staining

Clinical tissue samples were collected from 67 HCC patients from Beijing You’an Hospital of Capital Medical University between 2010 and 2015 (Supplementary Table 5), and procedures were performed with the approval of the institutional Ethics / Animal Committee. Paraffin sections (4 μm) were stained with hematoxylin and eosin (HE). After deparaffinization and rehydration, antigen retrieval was performed using the citric acid/pressure cooker method. Sections were washed with PBS and then incubated with NKCC-1 mouse monoclonal antibody (Proteintech group, USA) at 4°C overnight. Subsequently, sections were washed and incubated with the secondary antibody PV 6000 (universal antibody, Zhongshanjinqiao Biotech Company, Beijing, China) at 37°C for 10 min. After chromogenic staining with DAB, sections were dehydrated and mounted. Finally, the sections were reviewed under a microscope independently by two pathologists and blindly scored for NKCC1 immunoreactivity into four grades (-, 1+, 2+, and 3+) referencing plasma membrane protein HER2 scoring method [55].

### Knockdown and overexpression

For gene silencing, MHCC97H cell line was transfected with 100 pmol of shRNA or siRNA against NKCC1 or negative controls (Supplementary Table 6. synthesized by GenePharma, China). For gene overexpression, MHCC97L cell line was transfected with 4 μg of recombinant vectors (kindly provided by Dr. Biff Forbush, Yale University, New Haven, CT) containing NKCC1 gene. Transfection was performed using the lipofectamine^TM^ 2000 (Invitrogen) as instructed by the manufacture. Then the cells were cultured for 48 h. After G418 screening, the cell lines with stably lower or higher expression level of target proteins were obtained.

### *In vitro* analysis of tumor cell proliferation and invasion

Viability of cultured cells was measured by CCK-8 assay. For the matrigel cell invasion assay, transwell inserts containing 8 μM pore polycarbonate membranes were coated with matrigel, and the cells were allowed for invasion till 24 h. The invaded cells were visualized by hexamethyl pararosaniline staining. MMP-2 activity was detected by gelatin zymography. Details were shown in supplementary methods.

### *In vivo* animal studies

*In vivo* animal studies were performed with the approval of the institutional Ethics / Animal Committee. For the tumor xenograft analysis, either stable NKCC1 knockdown or overexpressed cells were injected subcutaneously into the flank of nude mice, and the tumor volumes were determined each week till 42 days. In addition, spleen and tail vein injection assays were performed to investigate intrahepatic metastasis and long-distance lung metastasis, respectively. The number of metastatic nodules was calculated, and the dissected tumors were histologically examined. To investigate the antitumor activities of bumetanide *in vivo*, the subcutaneous tumor-bearing mice were treated with bumetanide, sorafenib (positive control) or water (negative control) by gavage, followed by the measurement of tumor volume and weight. Details were shown in Supplementary Methods.

## SUPPLEMENTARY MATERIALS FIGURES AND TABLES









## Abbreviations

DTT: dithiothreitol
ECL: enhanced chemiluminescence
GAPDH: glyceraldehyde-3-phosphate dehydrogenase
GO: Gene Ontology
HEPES: 4-(2-hydroxyethyl)-1-piperazineethane sulfonic acid
LTQ-FT: linear ion trap quadrupole-fourier transform
MES: Methyl ethane sulfonate
PAGE: polyacrylamide gel electrophoresis
PBS: phosphate buffered saline
pH: hydrogen ion concentration
SD: standard deviation
SDS: sodium dodecyl sulfate
