# Supplementary material for: Discovery of NKCC1 as a potential therapeutic target to inhibit hepatocellular carcinoma cell growth and metastasis
Source: Oncotarget. 2017 Aug 12;8(39):66328–42. doi: 10.18632/oncotarget.20240 (PMC5630415; doi:10.18632/oncotarget.20240)
Supplement: Supplementary file 1 [file oncotarget-08-66328-s001.pdf]

Discovery of NKCC1 as a potential therapeutic target to inhibit hepatocellular carcinoma cell growth and metastasis

SUPPLEMENTARY MATERIALS

Analysis of tumor cell proliferation and invasion *in vitro*

Cell proliferation assay

Cells were incubated in 96-well plates (10^4 cells/well) for 12 h. Then 10 μL of CCK-8 solution was added to the medium and cultured for another 4 h. The absorbencies of each well, after rocking for 10 s, were read using a Thermo MK3 microplate reader at a wavelength of 450 nm. The curves for cell survival were drawn by Microsoft Excel 2003 software.

Matrigel cell invasion assay

Transwell inserts containing polycarbonate membranes with 8 μM pores were coated with Matrigel (BD Biosciences, Bedford, MA). After being rehydrated for 2 h in humidified tissue culture incubator in 37°C, 10^4 cells were added to the upper chamber and the lower chamber was filled with 0.8 mL of DMEM supplemented with FBS to induce chemotaxis. After 24 h of invasion, the noninvading cells in the upper chamber were removed. The invading cells on the undersurface of the membrane were fixed with 100% methanol and stained with1% hexamethylpararosaniline. Then, the invaded cells were counted under a microscope. Three invasion chambers were used for each group. Cell numbers from five fields were counted for each chamber.

MMP-2 activity detection

Cells were cultured in FBS-free high glucose DMEM for 24 h after 70–80% confluency had been reached. The supernatants were centrifuged at 300×*g* for 5 min and 2000×*g* for 10 min to remove the cell debris. After ultrafiltration with YM-3 Centrifugal Filter (Millipore, Billerica, MA, USA), the samples were loaded on 10% zymogram gelatin substrate gels for electrophoresis. Then gels were washed twice in renaturing buffer (Invitrogen) for 40 minutes at room temperature, followed by developing buffer washing for 40 min at room temperature and overnight incubation at 37°C. Gels were then rinsed by distilled water and stained with Coomassie brilliant blue. The proteolytic activity of MMP-2 was evidenced as clear band (zone of gelatin degradation) at 72 kDa against the blue background of stained gelatin.

In vivo animal studies

Tumorigenicity *in vivo*

Either stable NKCC1 knockdown or overexpressed cells ($2×10^6$) were injected subcutaneously into the flank of nude mice. Growth curves were plotted based on average tumor volume at the indicated time points. The tumor dimensions were measured, and the tumor volume was calculated: $V=\pi/6 \times$ larger diameter $\times$ (smaller diameter)^2. Tumor growth was observed for 6 weeks. The tumorigenic experiments were performed with six mice in each group.

Tumor metastasis *in vivo*

We employed spleen injection and tail vein injection assay to assess the effect of NKCC1 on tumor metastasis, using 6-7 weeks old BALB/c nude mice. The spleen injection was conducted to observe intrahepatic metastasis. The mice were anesthetized with 2.5% sodium pentobarbital (50 mg/kg, Sigma). Then the abdomen of the each mouse was opened and the upper part of spleen was injected with 200 μl of MHCC97H cells ($2×10^6$) stably transfected with shRNA or empty vector as control. Then the skin was closed. Mice were sacrificed 56 days after implantation. After macroscopic examination, the tumor was removed for hematoxylin and eosin (HE) staining and histologic examination. The pathologist was masked from the group designation of the samples. The liver weights were compared using the t-test.

In addition, $2×10^6$ of either stable NKCC1 knockdown or overexpressed cells were injected into the tail veins of BALB/c nude mice. The mice were observed for long-distance lung metastasis at 16 weeks after injection. The metastasis experiments *in vivo* were performed with six mice in each group.

In vivo bumetanide treatment experiment

MHCC97H cells were subcutaneously inoculated into the flank of nude mice. Water (negative control), 350 mg/kg of sorafenib (positive control), 1 mg/kg, 2 mg/kg and 4 mg/kg of bumetanide were administrated by oral gavage. After 18 days of administration, the tumor volume and weight in each mouse and the mouse body weight was measured.

Supplementary Table 1: Differential proteins

See Supplementary File 1

Supplementary Table 2: The effect of bumetanide on HCC growth *in vivo*

Group	Mice Number		Body Weight (g)		Tumor Size (mm^3)	Tumor Weight (g)	Tumor Inhibition Rate (%)	
	Start	End	Start	End			Size	Weight
Control	8	8	21.2±2.2	21.7±2.2	625.8±450.4	0.66±0.30	-	-
Positive Control	8	8	21.6±1.9	21.3±2.1	225.0±163.4*	0.24±0.17**	64.0	64.0
1 mg/kg	8	8	22.0±0.9	22.3±1.3	541.3±444.7	0.51±0.46	13.5	22.2
2 mg/kg	8	8	21.3±1.1	21.3±3.3	379.4±294.3	0.37±0.35	39.4	43.9
4 mg/kg	8	8	20.4±1.4	18.3±2.5*	389.0±261.8	0.29±0.24*	37.8	55.8

*p<0.05 or **p<0.01 indicates a significant difference of two-tailed Student's t-test.

Supplementary Table 3: Corrlation between expression of glypican-3, keratin 19 or Ki-67 and clinical features in HCC

See Supplementary File 2

Supplementary Table 4: Abs for Western Blotting

Antibody	Company/Provider	Species	Clonality	WB Dilution
alcohol dehydrogenase	Abcam	rabbit	poly	1:1000
flotillin-1	BD Biosciences	mouse	mono	1:1000
GAPDH	Proteintech	mouse	poly	1:2000
KDEL (Lys-Asp-Glu-Leu)	Stressgen	mouse	mono	1:1000
lamin B	Santa Cruz Biotechnology	goat	poly	1:200
NKCC1	Developmental Studies Hybridoma Bank	mouse	mono	1:500
OSR1	Abcam	mouse	mono	1:1000
phospho-NKCC1 (p-NKCC1)	Dr. Biff Forbush's lab	rabbit	poly	1:3000
phospho-OSR1 (Ser325, p-OSR1)	Dr. Hiroshi Shibuya's lab	rabbit	poly	1:200
phospho-SPAK (Ser373, p-SPAK)	Dr. Hiroshi Shibuya's lab	rabbit	poly	1:200
phospho-WNK1 (p-WNK1)	R&D Systems	rabbit	poly	1:200
SPAK	Millipores	mouse	mono	1:1000
WNK1	Santa Cruz Biotechnology	rabbit	poly	1:100
β-actin	Abcam	mouse	mono	1:1000

Supplementary Table 5: Clinical samples

See Supplementary File 3

Supplementary Table 6: shRNAs

shRNA / siRNA	Target sequence
human NKCC1 shRNA-#1	5'-GCGTTAATGACACTATCGTAT-3'
human NKCC1 shRNA-#2	5'-GCGATTTAGATACTTCCAAAT-3'

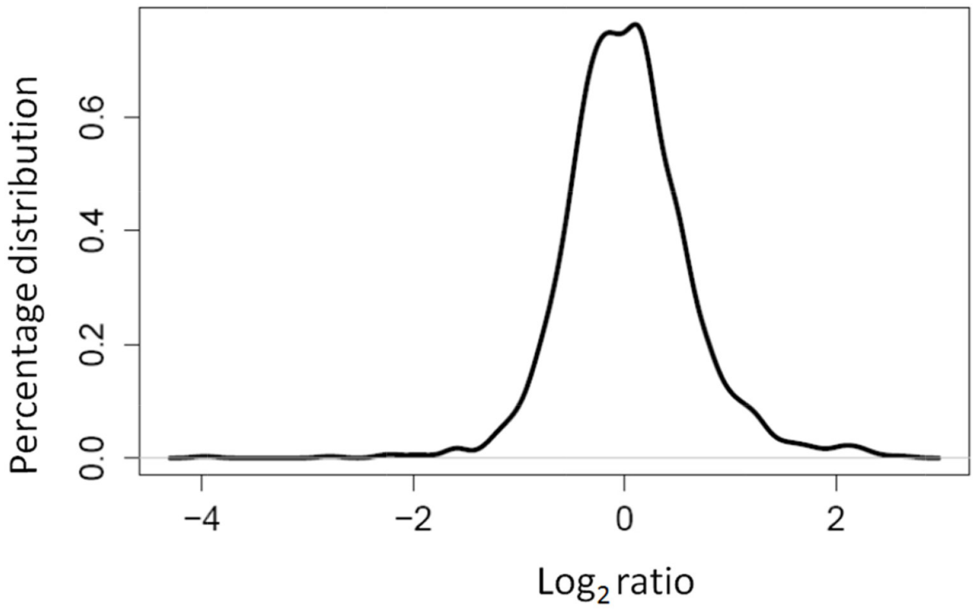

Supplementary Figure 1: Ratio distribution of all the proteins quantified by SILAC in MHCC97H and MHCC97L cells.

Peptide A: AAAAAAAAAAAAAAAAGAGAGAk

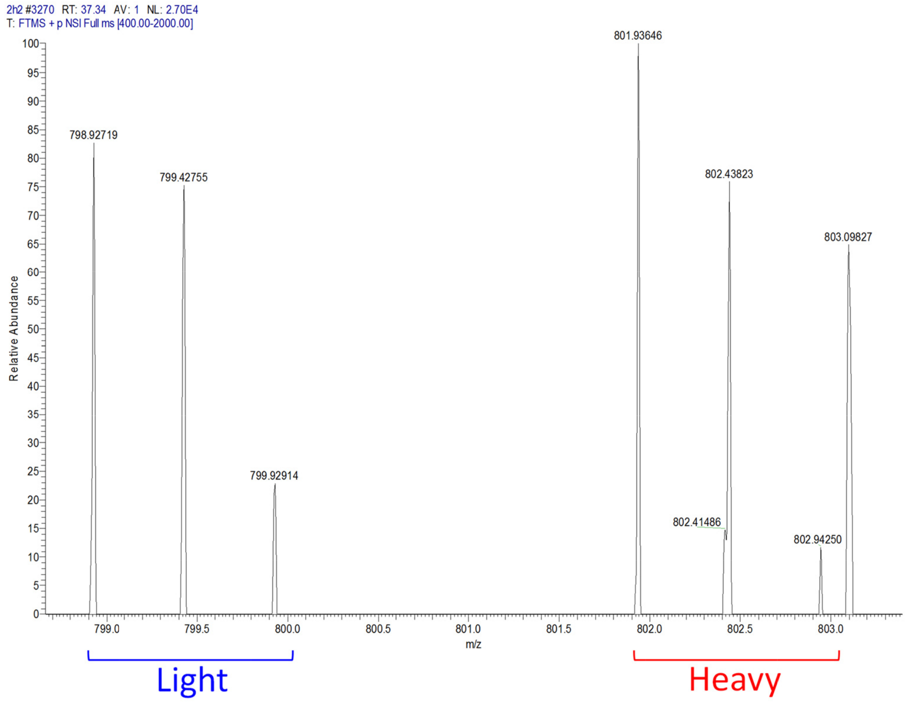

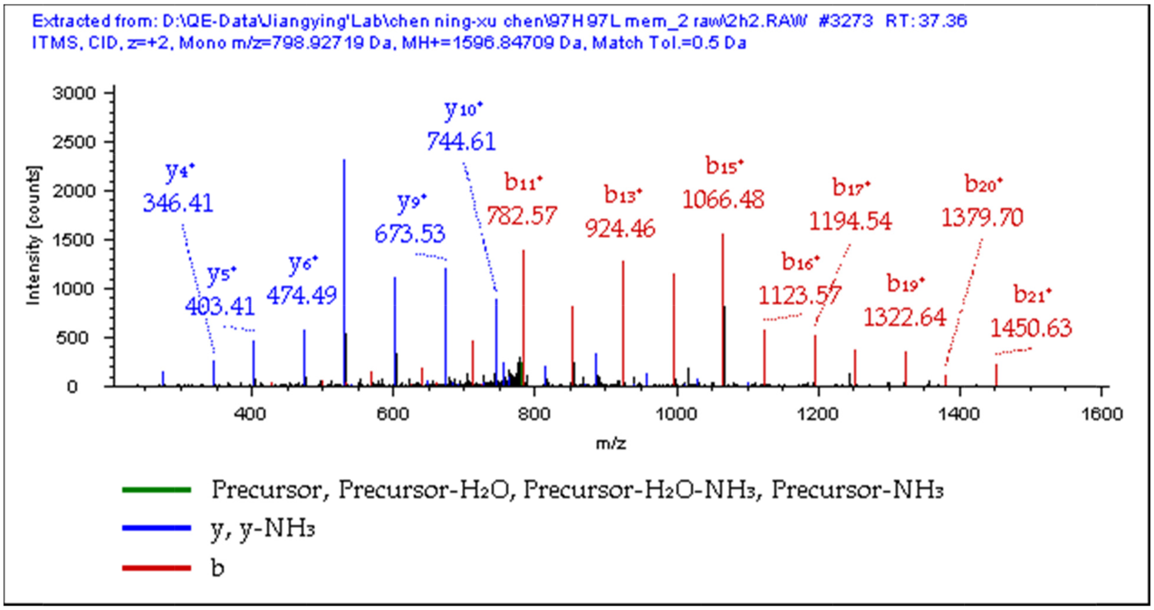

(*Continued*)

Peptide B: GGGAYYLISR

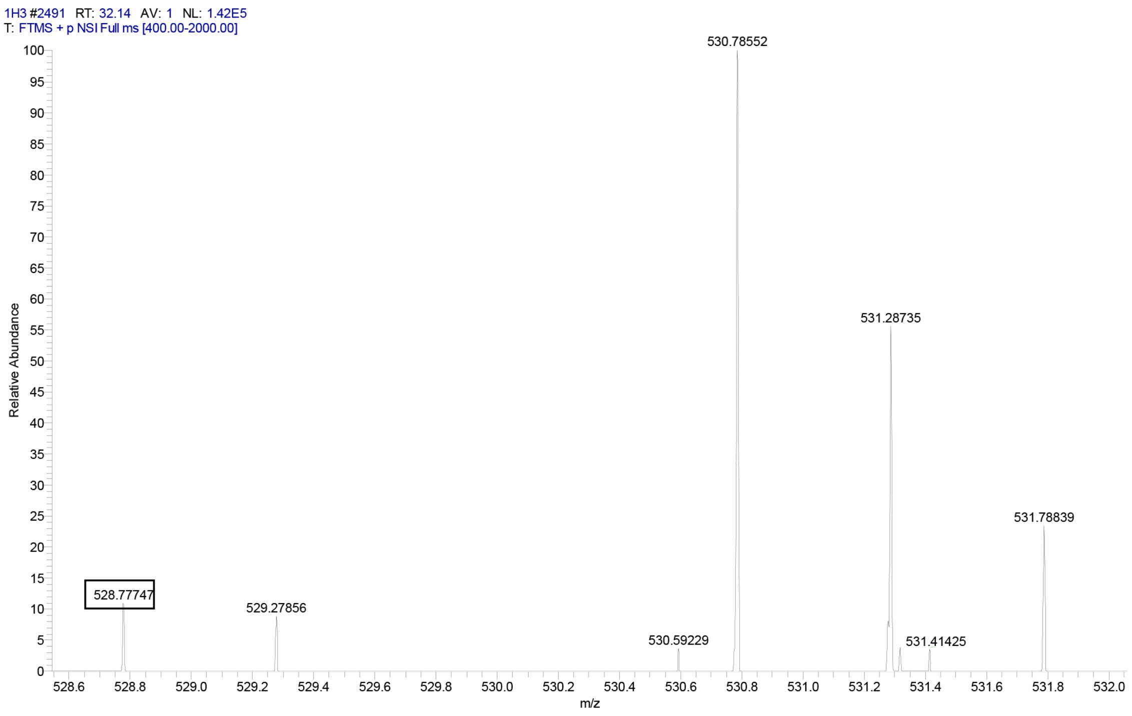

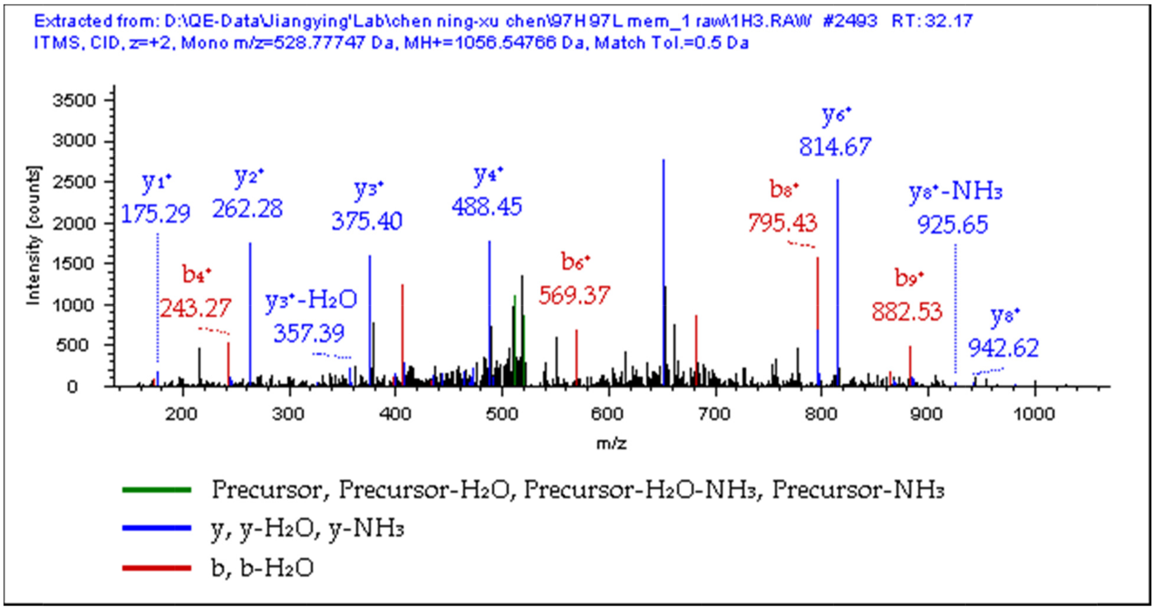

(*Continued*)

Peptide C: VELPGTAVPSVPEDAAPASR

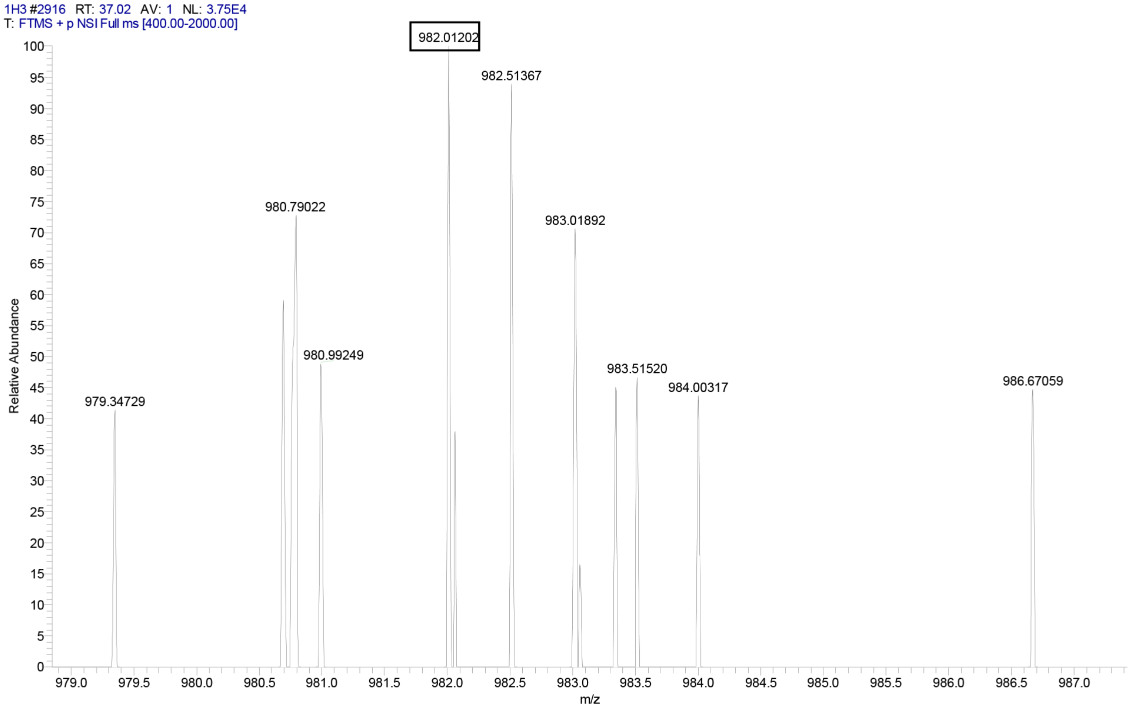

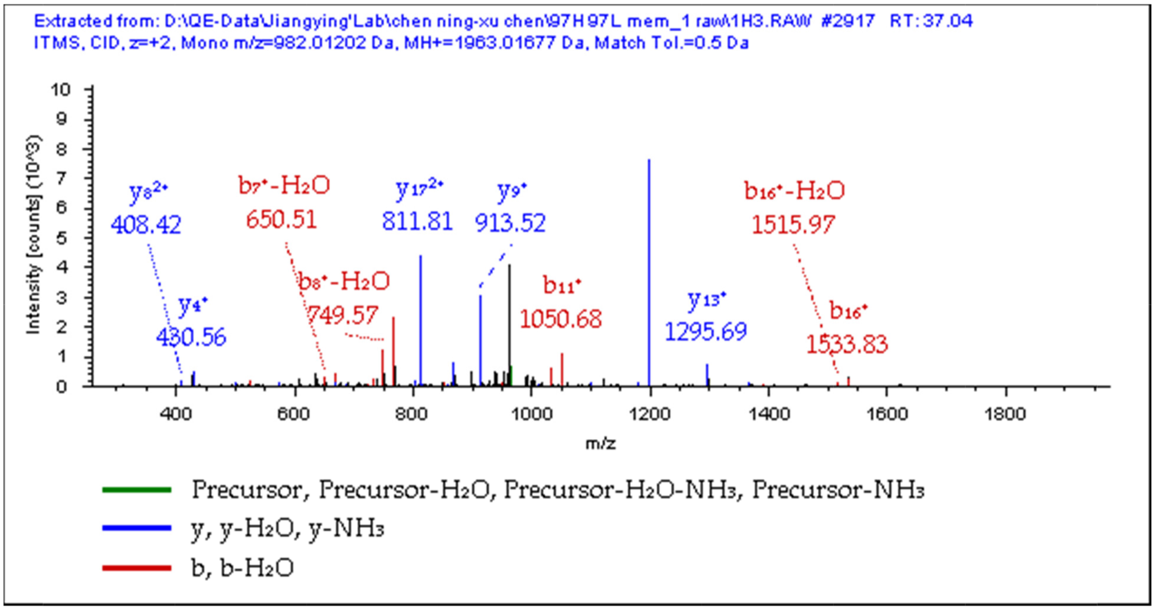

(*Continued*)

Peptide D: FQVDLVSENAGR

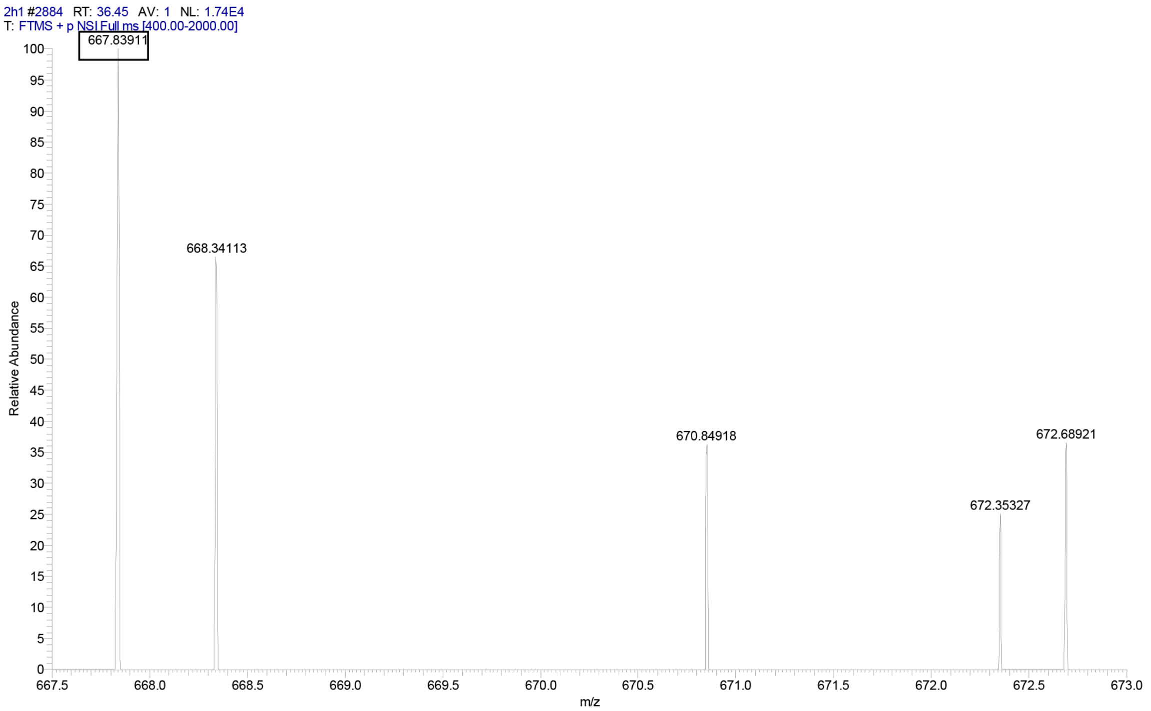

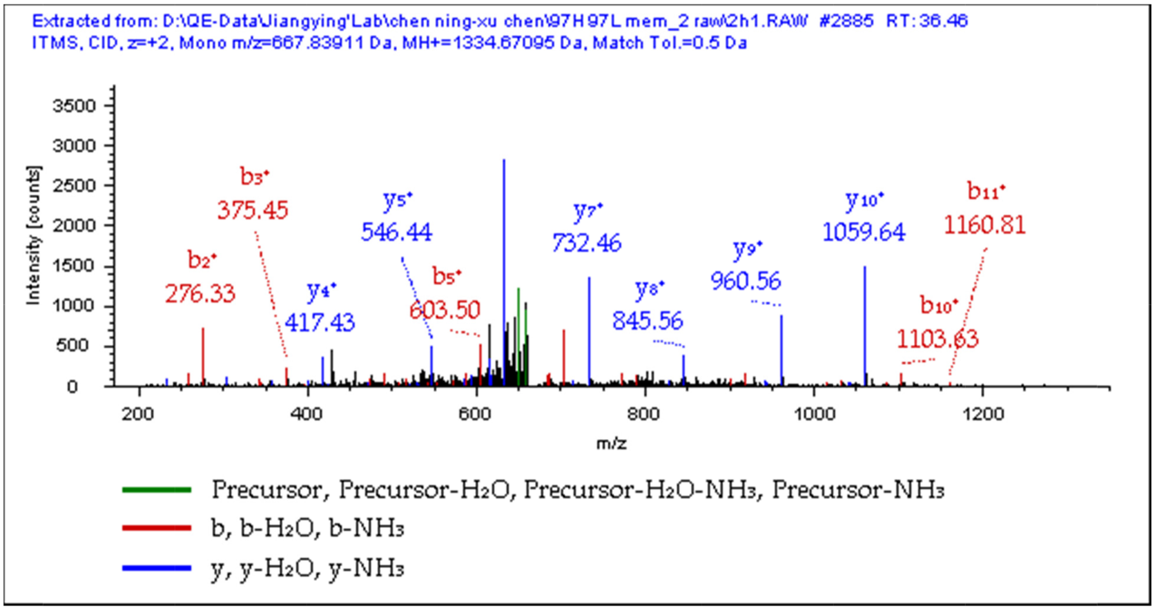

(*Continued*)

Peptide E: AFYAPVHADDLREGAQYLMQAAGLGR

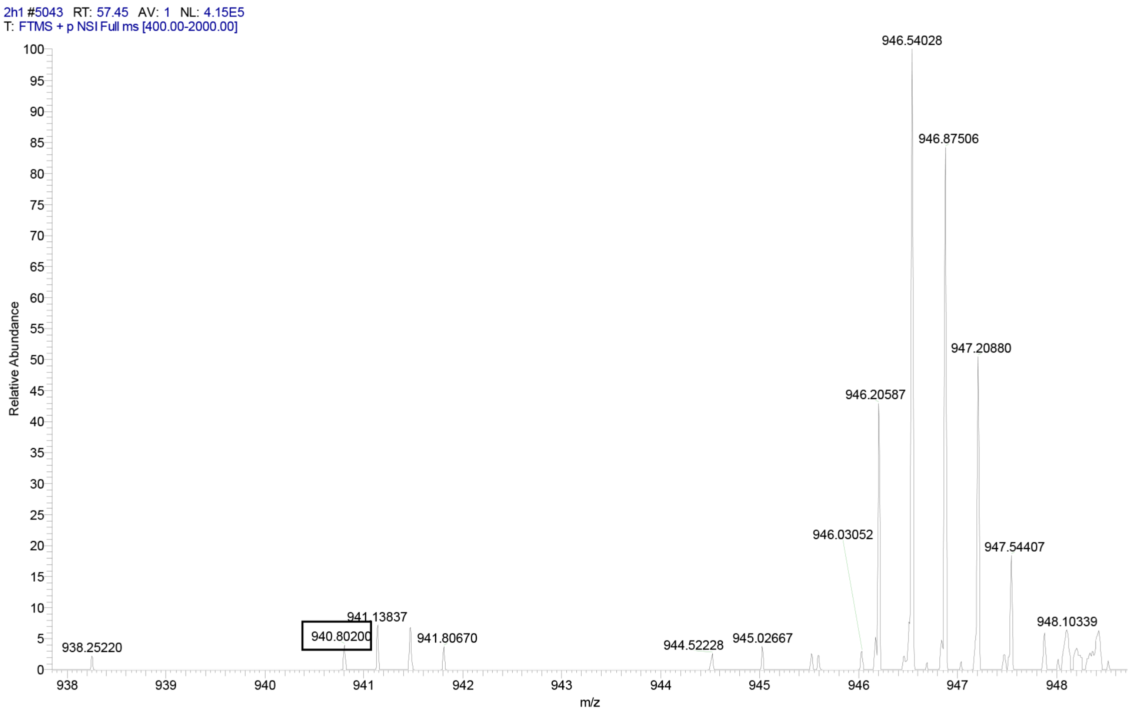

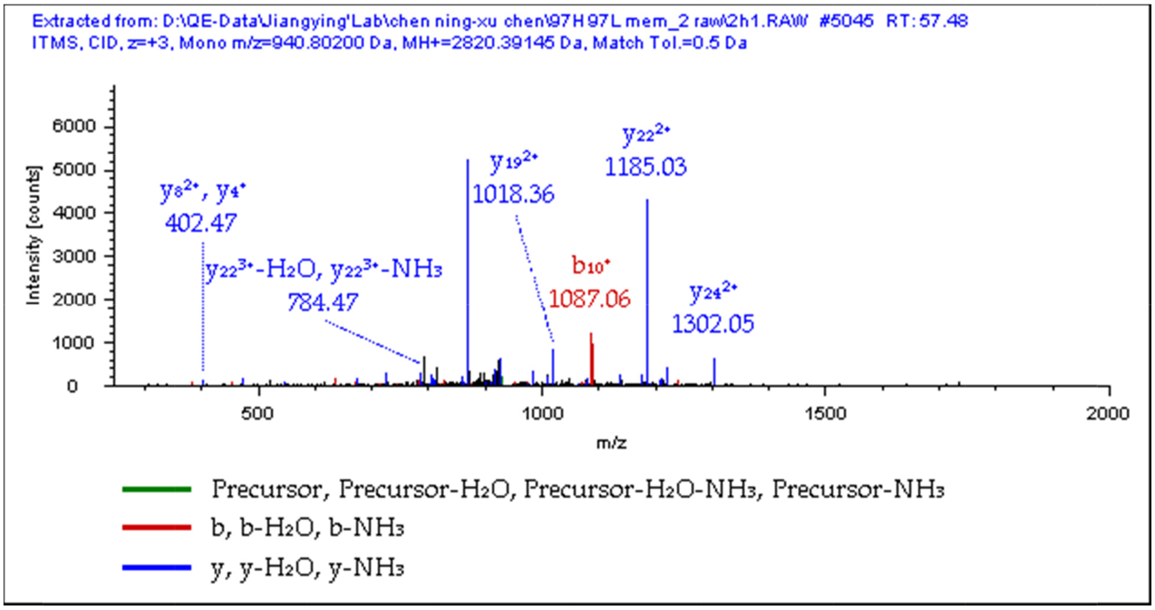

Supplementary Figure 2: The MS (the parent ion was marked with rectangle) and MS/MS spectrum of five peptides **(A-E)** identified in NKCC1. Peptide A was used for quantification. Peptide ratio was determined by calculating the areas under the monoisotopic peaks of heavy isotopic ($^{13}C_6$-lysine, red arrows) versus light isotopic ($^{12}C_6$-lysine, blue arrows) peptides.

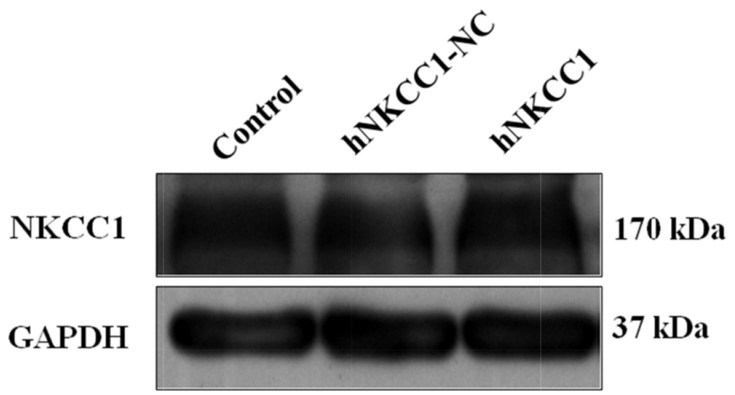

Supplementary Figure 3: NKCC1 overexpression in MHCC97L. Exogenous NKCC1 was overexpressed in MHCC97L cell transfected with the pcDNA3.1 vector. Parental cells with empty vector were used as a control.

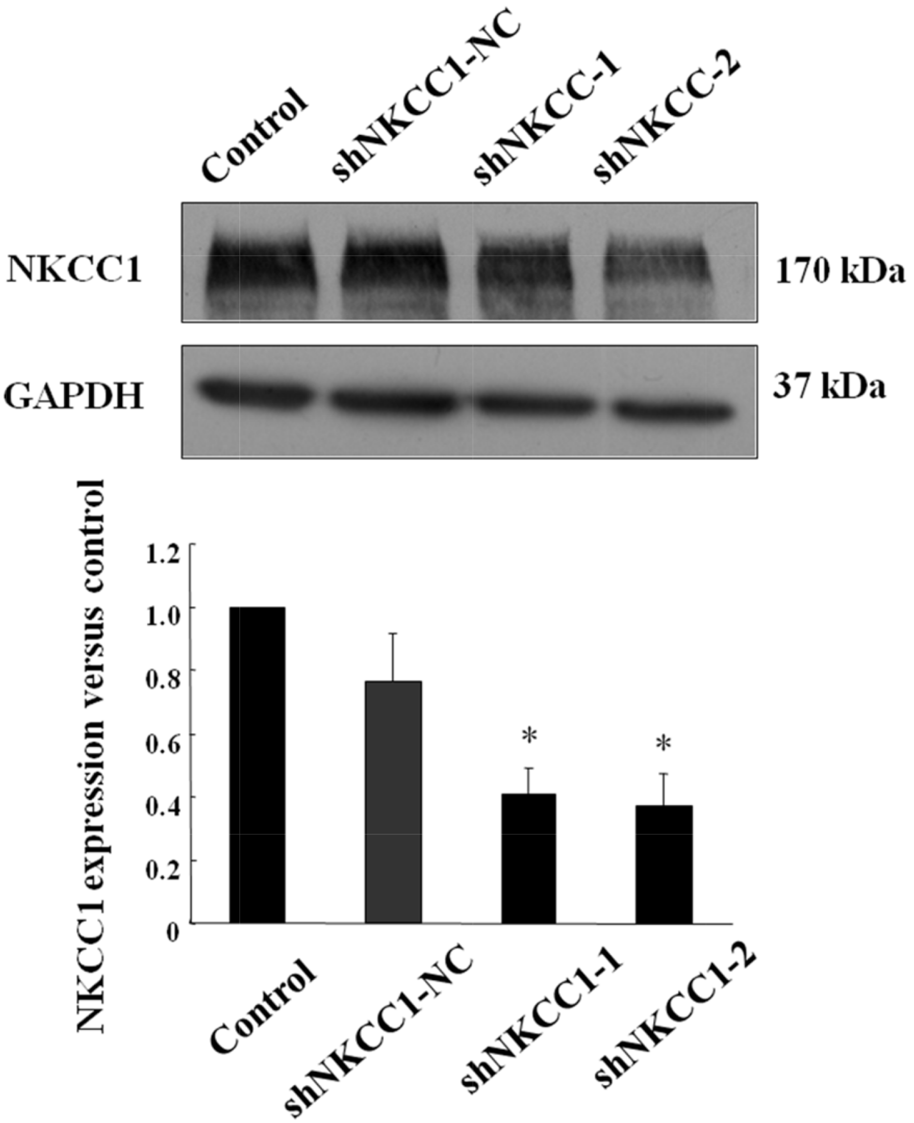

Supplementary Figure 4: shRNA knockdown of NKCC1 in MHCC97H. Relative protein (upper) and mRNA (lower) level of NKCC1 was reduced after shRNA transfection in MHCC97H. * $p<0.05$ indicates a significant difference (one-way ANOVA).

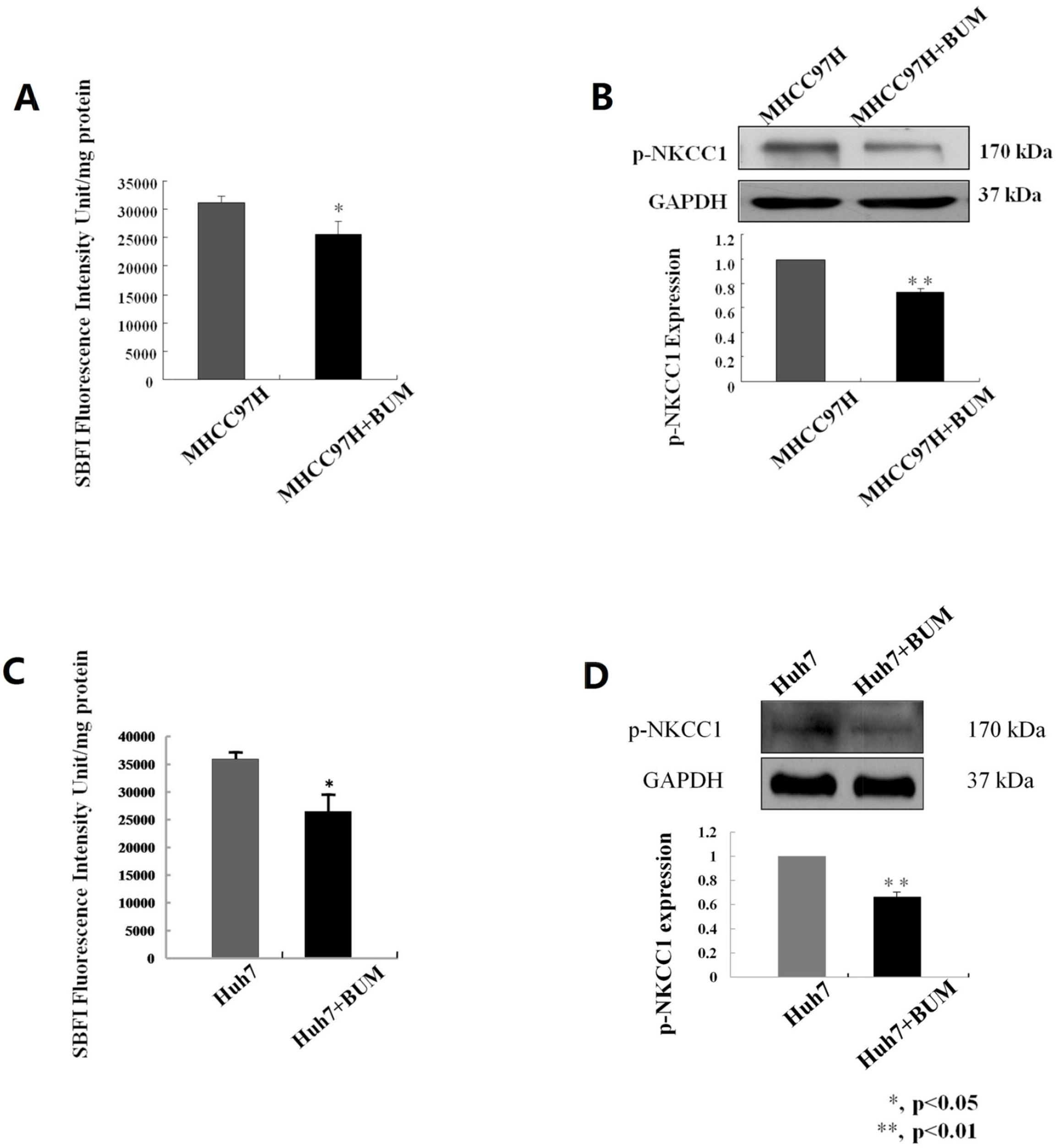

Supplementary Figure 5: Bumetanide treatment inhibited the activity and phosphorylation level of NKCC1 in MHCC97H cells and Huh7 cells *in vitro*. MHCC97H and Huh7 were exposed to 50 µM bumetanide. Phospho-NKCC1 levels were normalized against GAPDH. **(A)** Fluorescence intensity measurement of enzyme activity showed that bumetanide treatment inhibited NKCC1 activity in MHCC97H cells. **(B)** Bumetanide treatment inhibited NKCC1 phosphorylation in MHCC97H cells. **(C)** Fluorescence intensity measurement of enzyme activity showed that bumetanide treatment inhibited NKCC1 activity in Huh7 cells. **(D)** Bumetanide treatment inhibited NKCC1 phosphorylation in Huh7 cells. * $p<0.05$ or ** $p<0.01$ indicates a significant difference (two-tailed Student's *t*-test).

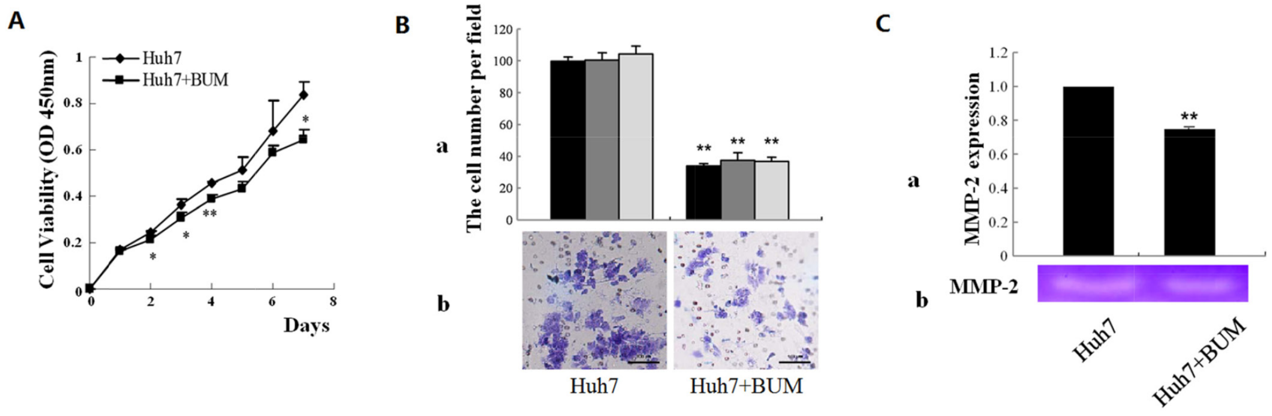

Supplementary Figure 6: Bumetanide treatment inhibited the cell growth, invasion and MPP-2 activity in Huh7 cells *in vitro*. (A) CCK-8 kit analysis shows that Huh7 cell proliferation was significantly inhibited following bumetanide treatment. **(B)** The matrigel assay shows that the invasion of Huh7 cells was significantly inhibited following bumetanide treatment. (B-a) Counts of trespassed cells per field (from at least five fields) from three experiments (mean±SD). (B-b) Representative photographs after 24-h incubation. **(C)** The activity of MMP-2 in Huh7 cells was significantly inhibited following bumetanide treatment. (C-a) Statistical results of triplicate experiments. (C-b) Representative photographs. * p<0.05 or ** p<0.01 indicates a significant difference between bumetanide treatment with the normal control group (two-tailed Student's t-test).

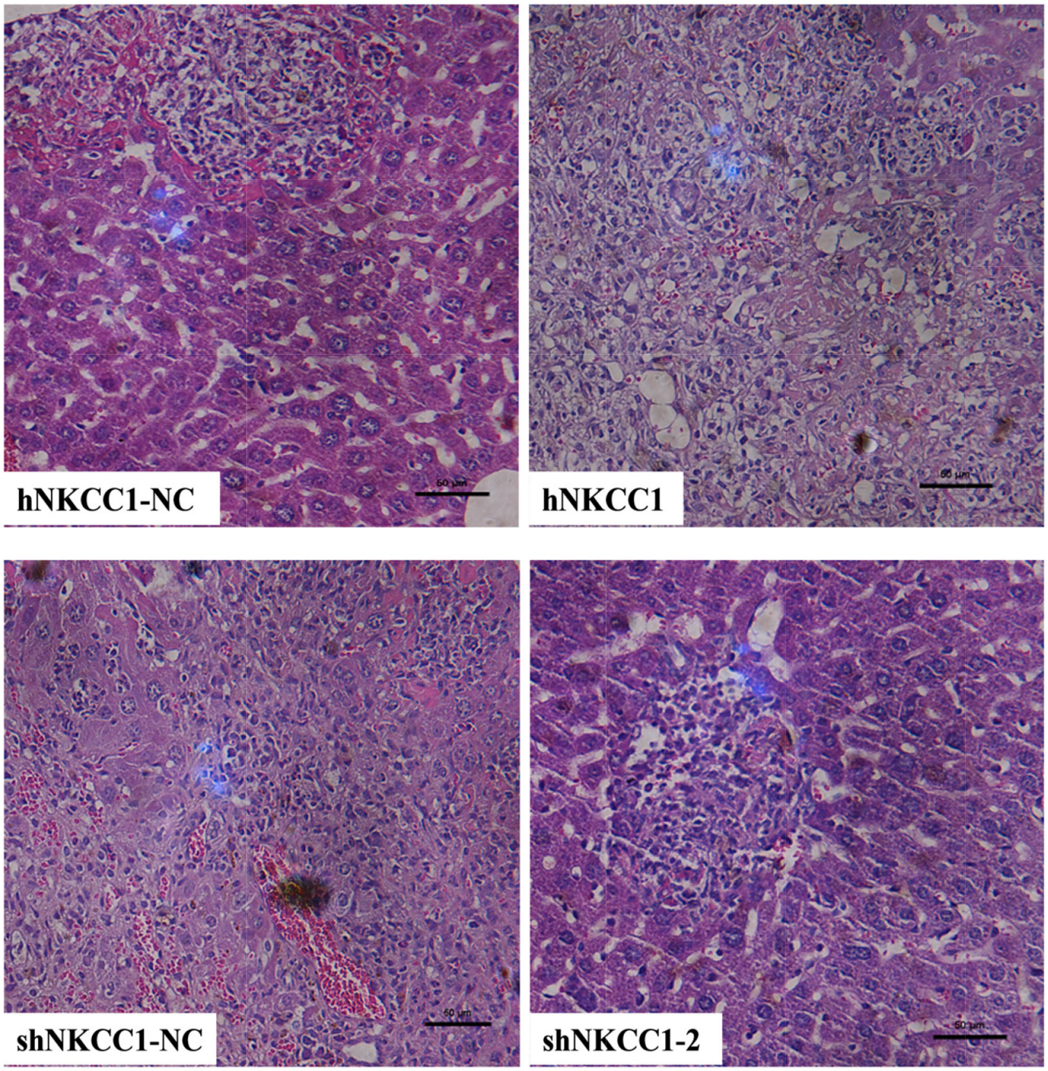

Supplementary Figure 7: The *in vivo* effect of NKCC1-overexpression on HCC intrahepatic metastasis after tail vein inoculation. Representative images of metastases that formed in the livers at 16 weeks after tail vein injection with NKCC1-overexpress MHCC97L cells (up-right) and control cells (up-left), or NKCC1-knockdown MHCC97H cells (down-right) and control cells (down-left).

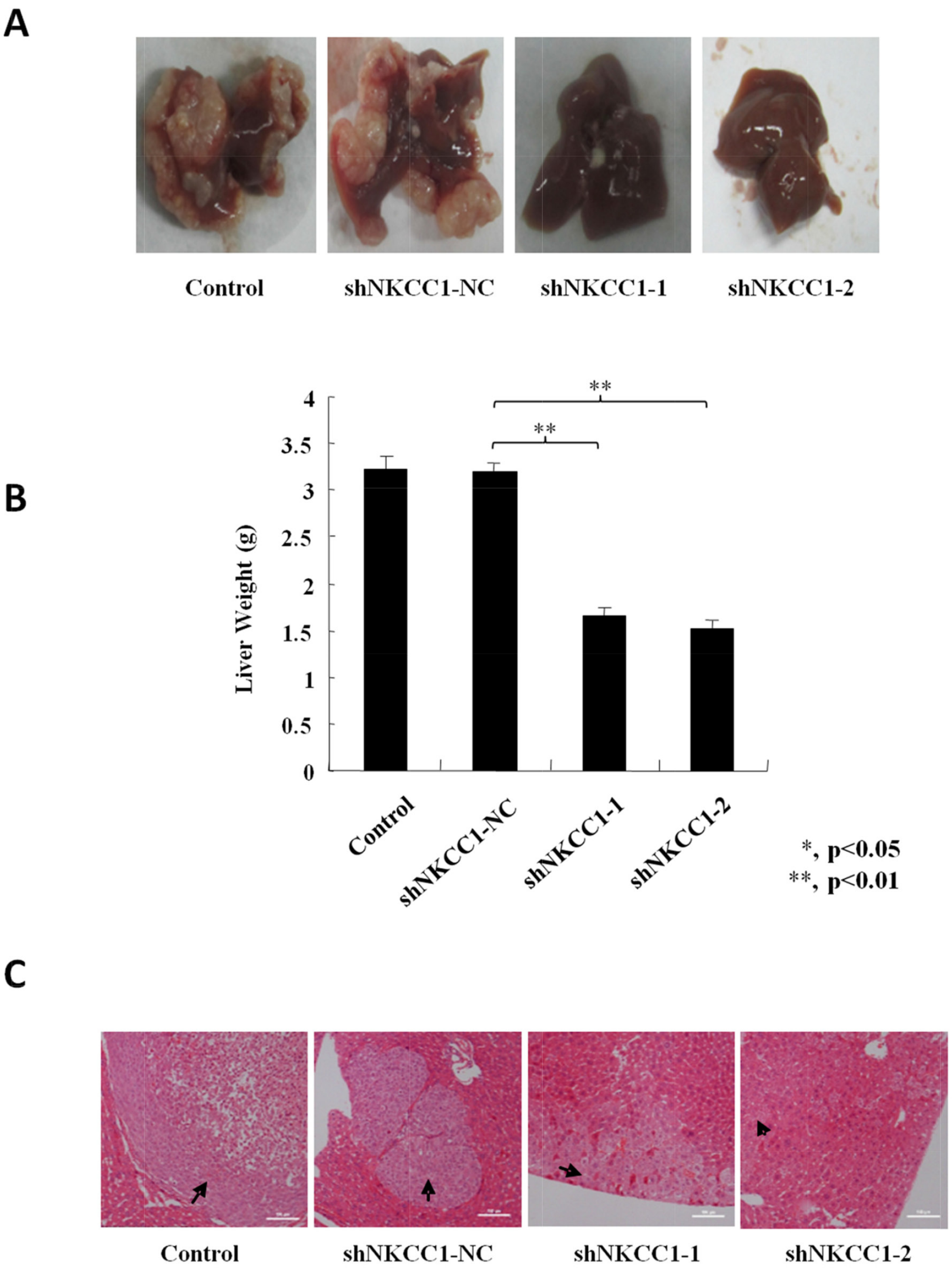

Supplementary Figure 8: The *in vivo* effect of NKCC1-knockdown on HCC intrahepatic metastasis after spleen inoculation. **(A)** Stable NKCC1-knockdown MHCC97H cells were injected into the spleen of BALB/c nude mice. After 8 weeks, obvious liver metastatic nodules could be seen in mice inoculated with MHCC97H cells transfected with shRNA-NC (representative images show the livers). **(B)** The total liver weights (mean±SD) were significantly decreased in shRNA-NKCC1 groups than in shRNA-NC group. * $p<0.05$ or ** $p<0.01$ indicates a significant difference (one-way ANOVA). **(C)** Histological examination confirmed the presence of liver tumors in these mice.
